# Supplementary material for: GSTZ1‐1 Deficiency Activates NRF2/IGF1R Axis in HCC via Accumulation of Oncometabolite Succinylacetone
Source: EMBO J. 2019 Jun 28;38(15):e101964. doi: 10.15252/embj.2019101964 (PMC6669923; doi:10.15252/embj.2019101964)
Supplement: Supplementary file 1 — Appendix [file EMBJ-38-e101964-s001.pdf]

GSTZ1-1 Deficiency Activates NRF2/IGF1R Axis in HCC via Accumulation of Oncometabolite Succinylacetone

Fan Yang[1,2,#], Jingjing Li[1,#], Haijun Deng[3,#], Yihao Wang[4], Chong Lei[1], Qiujie Wang[1], Jin Xiang[1], Li Liang[1], Jie Xia[1], Xuanming Pan[1], Xiaosong Li[2], Quanxin Long[1], Lei Chang[4], Ping Xu[4], Ailong Huang[1,*], Kai Wang[1,*], Ni Tang[1,*]

Table of Contents

Appendix Supplementary Methods......2

Appendix Figure S1......16

Appendix Table S1......17

Appendix Table S2......21

Appendix Table S3......23

Appendix Supplementary Methods

GEO Data Analysis

GSE5975 dataset (Gene Expression Profiling of Hepatocellular Carcinoma) was downloaded from Gene Expression Omnibus (GEO) online. To identify the differentially expressed genes (DEGs) between paired HCC and para-carcinoma tissue in EpCAM positive and EpCAM negative groups, the differential expression analysis for the mRNA was performed, we used log2 fold change greater than 2 as filtering for mRNA. To interpret biological implications of the DEGs, we carried out Kyoto Encyclopedia of Genes and Genomes (KEGG) metabolism pathway enrichment analysis, using the KOBAS3.0 program under a significance threshold of $P < 0.05$ (http://kobas.cbi.pku.edu.cn/index.php).

TCGA Data Analysis

Gene expression data of *GSTZ1* and *IGF1R* were obtained from The Cancer Genome Atlas (TCGA) Liver Hepatocellular Carcinoma (LIHC) dataset. To compare expression levels, we used the reads per kilobase per million mapped reads (RKPM) or RNA-sequencing (RNA-seq) by expectation maximization (RSEM), two RNA-seq normalization methods for describing transcript abundance of genes. In the box-scatterplot, boxes represent the two quartiles around the median. Kaplan-Meier survival analysis was performed by patient stratification of *GSTZ1* mRNA expression, high (> 25 percentile) or low (≤ 25 percentile).

Quantitative RT-PCR (qRT-PCR)

Total RNA was extracted from cell lines and frozen tumor specimens using TRIzol™ reagent (Invitrogen, Carlsbad, CA, USA) and reverse transcribed into cDNA using a PrimeScript™ RT reagent kit (Takara, Shiga, Japan) according to the manufacturer's instructions. Quantitative PCR was performed using iTaq™ Universal SYBR® Green Supermix and CFX

Connect Real-time PCR Detection System (Bio-Rad, Hercules, CA, USA). The constitution of the 10 μl PCR reaction system was as follows: 2 μl cDNA, 0.5 μl forward primer (10 mM), 0.5 μl reverse primer (10 mM), 5 μl iTaq™ Universal SYBR® Green Supermix (Bio-Rad), and 2 μl nuclease-free water. The reaction conditions were as follows: one cycle at 95°C for 30 seconds, followed by 35 cycles of amplification at 95°C for 10 seconds, then at 62°C for 30 seconds, and then at 72°C for 30 seconds. All samples were run in triplicate in each experiment. The objective CT values were normalized to that of actin, and relative gene expression was calculated using the $^{\Delta\Delta}$CT method. The related primers were listed in Appendix Table S1.

Western Blotting (WB)

Total protein was extracted from cells or tissues using RIPA Lysis Buffer (Strong) (Cwbiotech, Beijing, China) containing 1 mM phenylmethylsulfonyl fluoride (PMSF; Beyotime, Shanghai, China). Cytoplasmic and nuclear cellular proteins were extracted using a Nuclear and Cytoplasmic Protein Extraction Kit (Beyotime) according to the manufacturer's instructions. Protein concentrations were measured using a BCA protein assay Kit (Dingguo, Beijing, China). Protein samples were separated by 8-12.5% sodium dodecyl sulfate polyacrylamide gel electrophoresis (SDS-PAGE) at 90-120 V for 90-120 minutes, and then transferred to polyvinylidene difluoride (PVDF) membranes (Millipore, Boston, MA, USA). The membranes were blocked in 5% nonfat dry milk, or in bovine serum albumin in tris-buffered saline and Tween-20 (TBS-T; 10 mM Tris, 150 mM NaCl, and 0.1% Tween-20). Then, the membranes were incubated with following primary antibodies, diluted in bovine serum albumin, overnight at 4°C. Primary antibodies included: GSTZ1-1, IGF1Rβ, phospho (Tyr1131) IGF1Rβ, NQO1, TXNRD1, HO1, NRF2, Myc, Flag, AKT, phospho (Thr308) AKT, ERK, phospho (Thr202/Thr204) ERK, phospho (Ser136) BAD, phospho (Ser133) CREB, BCL2, caspase9,

and caspase3. The membranes were washed three times in TBS-T followed by incubation with secondary antibodies conjugated with horseradish peroxidase (HRP), for 1 hour at room temperature. The membranes were washed three additional times with TBS-T before enhanced chemiluminescence exposure. The expression of actin, tubulin, and laminB1 were used as normalization controls for total, cytoplasmic, and nuclear proteins, respectively. Antibody information was showed in Appendix Table S2.

Immunohistochemical (IHC) Assay

Human or mouse liver tissue samples were fixed in 4% paraformaldehyde and embedded in paraffin according to standard procedures. The paraffin sections were incubated at 57°C for 2-4 hours, de-paraffinized in xylene for 20 minutes, and successively rehydrated in 100, 95, 85, and 75% ethanol, and in distilled water. Samples were boiled in citrate buffer and cooled down at room temperature for antigen retrieval. Samples were then incubated with 0.5%Triton X-100 for 20 minutes, followed by incubation with 3% hydrogen peroxide for 10 minutes. Samples were then washed with phosphate buffered saline (PBS) three times and blocked with goat serum for 1 hour at room temperature. Next, the serum was removed, and the samples were incubated with the following primary antibodies, diluted in PBS, overnight at 4°C. Primary antibodies included: GSTZ1-1, IGF1Rβ, and phospho (Tyr1161) IGF1Rβ. After rinsing three times in PBS, samples were incubated with reagent A of Elivision™ plus Polyer HRP (Mouse/Rabbit) IHC Kit (Maixin, Fuzhou, Fujian, China) for 20 minutes, followed by incubation with reagent B, and then with secondary antibody conjugated to HRP for 30 minutes. Signals indicating HRP activity were visualized using a DAB Kit (ZSGB-BIO, Beijing, China). The nuclei were counterstained with hematoxylin for 2 minutes and 30 seconds. After being rinsed, the samples were dehydrated, cleared in xylene, and mounted for microscopic examination. Antibody information was showed in Appendix Table S2.

Cell Culture

The human hepatoma cell line HepG2 was obtained from the American Type Culture Collection (ATCC, Manassas, VA, USA). Huh7, SK-Hep1, MHCC-97H and HEK293T were obtained from the Cell Bank of the Chinese Academy of Sciences (Shanghai, China). Cells were maintained in Minimum Eagle medium (MEM for HepG2; Hyclone, Waltham, MA, USA) or Dulbecco's modified Eagle's medium (DMEM for all other cells; Hyclone) supplemented with 10% fetal bovine serum (FBS; Natocor, Villa Carlos Paz, Córdoba, Argentina) and 1% penicillin/streptomycin (Hyclone) in 5% CO_2 incubator at 37°C.

GSTZ1-1 overexpression in HCC cell lines

Full-length *GSTZ1* cDNA (NM_145870.2) was amplified from the plasmid pOTB7-GSTZ1-1 (FL09522; GeneCopoeia, Guangzhou, Guangdong, China) and inserted into the *BamH I* and *Hind III* sites of the shuttle vector pAdTrack-TO4 (kindly provided by Dr. T-C He, University of Chicago, USA). A recombinant adenovirus expressing GSTZ1-1 (AdGSTZ1) was generated using the AdEasy system (Luo et al, 2007). Huh7, SK-Hep1 and MHCC-97H cells, which endogenously express low levels of GSTZ1-1, were infected with AdGSTZ1 to establish the GSTZ1-1 overexpressing (GSTZ1-OE) cell model. An analogous adenovirus expressing green fluorescent protein (AdGFP) was used as control.

CRISPR-Cas9 mediated *GSTZ1* knockout in HepG2 cell line

The *GSTZ1* targeting sequences (Appendix Table S1) were designed using the E-CRISP online tool (http://www.e-crisp.org/E-CRISP/designcrispr.html) and cloned into a lentiCRISPRv2 plasmid following the protocol established in the Zhang laboratory (http://genome-engineering.org). lentiCRISPRv2 was digested with *BsmB I*, ligated with the

annealed oligonucleotides, and transformed into DH10B competent bacteria. To package the lentivirus (Lenticrispr-GSTZ1-1), the recombinant plasmid was co-transfected into HEK293T cells with the packaging plasmids pGMD and psPAX2 using Lipofectamine 2000 (Invitrogen) per manufacturer's protocol. HepG2 cells were infected with the lentivirus and selected using puromycin to establish a stable *GSTZ1* knockout (GSTZ1-KO) cell line. Single-cell clones were then selected using a 96-well plate. For genotyping, clonal cell genomic DNA was extracted using a Genomic DNA Purification Kit (Genloci, Nanjing, Jiangsu, China) according to the manufacturer's instructions. The genomic region flanking the small guide RNA (sgRNA) target site was amplified by touchdown PCR, cloned into an pMD19-T TA cloning vector (Takara, Kyoto, Japan), and sequenced. The efficiency of *GSTZ1* knockout was confirmed by western blotting.

EdU Assay

The proliferation capacity of HCC cells was assessed using a Cell-Light™ EdU Apollo®643 Cell Tracking Kit (RiboBio, Guangzhou, Guangdong, China), according to the manufacturer's instructions. Cells were seeded on coverslips in 6-well plates and grown to 50% confluence using three replicate wells for each group. GSTZ1-KO cells were treated with or without picropodophyllin (PPP; MCE, Monmouth, NJ, USA) (1.0 μM) for 24 hours. Cells were then incubated with Reagent A diluted at 1:1000 in culture medium for 2 hours. The cells were then washed using PBS, fixed in 4% paraformaldehyde for 30 minutes, and neutralized in 2 mg/ml glycine solution. Then cells were then incubated with a fluorescent dye (Apollo 643) for 30 minutes, and consequently wash with 0.5% Triton X-100 in PBS in the dark. Next, the nuclei were stained with 1 μg/ml DAPI (Roche, Mannheim, Germany) for 2 minutes and then washed in PBS. The coverslips were mounted on glass slides using Anti-fade Mounting Medium (Beyotime). Images were captured using a confocal microscope

(Leica, Buffalo Grove, IL, USA). The percentage of 5-ethynyl-2'-deoxyuridine (EdU)-positive cells was calculated for comparison.

Cell Growth Curve and Clone Formation Capacity

Cells were seeded into 96-well plates (1500 cells/well for the GSTZ1-OE cell model and 3000 cells/well for the GSTZ1-KO cell model) using three replicate wells for each group. GSTZ1-KO cells were treated with or without PPP (0.5 μM and 1.0 μM) for 24 hours. The cells were incubated in CelCulture® CO_2 Incubator (ESCO, Changqi South Street, Singapore) for 120 hours, and the number of cells was counted automatically every 24 hours. Growth curves were plotted using GraphPad Prism 7 software.

Cells were seeded into 6-well plates (1500 cells/well for the GSTZ1-OE cell model and 3000 cells/well for the GSTZ1-KO cell model) using three replicate wells for each group, and were cultured in 5% CO_2 incubator at 37°C. After 7-10 days, the cells were fixed with 4% paraformaldehyde for 30 minutes and incubated with crystal violet. After rinsed with PBS three times, the clones were photographed and the number of clones was counted.

Dual-Luciferase Reporter Assay

The promoter activity of antioxidative response element (ARE) and human IGF1R were detected using Dual-Luciferase Reporter System (Promega, Madison, WI, USA). The ARE reporter plasmid pGL3-ARE were kindly provided by Prof. Yiguo Zhang from Chongqing University. The IGF1R promoter was synthesized and cloned into a pGL3 plasmid by Tsingke Biological Technology (Beijing, China). The plasmids, pGL3-ARE (3 μg) or pGL3-IGF1R (3 μg) were co-transfected with RL-TK (0.3 μg), into cells cultured in 6 cm dishes. After 24 hours, the cells were re-seeded into 24-well plate, three replicate wells for each group. 36 hours later, the cells in the 24-well plates were lysed with 1×Passive Lysis

Buffer for 15 minutes on a shaker at room temperature. The lysates were centrifuged, and the supernatants were collected. The activities of firefly and Renilla luciferases were detected according to the manufacturer's instruction. The relative luciferase activities, which represented the promoter activities, were calculated by normalizing the activity of firefly luciferase to that of corresponding Renilla luciferase.

Xenograft Implantation Model in vivo

The male nude mice aged 4 weeks were obtained from Experimental Animal Center of Chongqing Medical University. MHCC-97H cells were infected with GSTZ1-1 expressing adenovirus (AdGSTZ1) or a control adenovirus expressing GFP (AdGFP) for 36 hours. Then the cells were digested by trypsin. 1×10^6 cells were resuspended in 100 μl of PBS and were inoculated subcutaneously into either side of the dorsal flank of the nude mouse. Each group included six mice. The tumor volumes were measured every three days once the tumors were formed, and calculated as length × width × height. After 4-5 weeks, the mice were sacrificed and the tumors were carefully removed and weighed. All animal procedures were performed according to protocols approved by the Rules for Animal Experiments published by the Chinese Government and approved by the Research Ethics Committee of Chongqing Medical University (reference number: 2017010).

Immunofluorescence (IF) Assay

Cells, infected with AdGSTZ1 or AdGFP (control), were seeded on coverslips in 6-well plate. The cells were fixed with 4% paraformaldehyde for 25 minutes, and then permeabilized with 0.5% Triton X-100 for 15 minutes. After washed by 1×PBS, the cells were blocked with goat serum for 1 hour, followed by overnight incubation at 4°C with anti-NRF2 primary antibody (1:300, Abcam, ab62352) in 1×PBS. After that, the excess antibody was washed off with

1×PBS. Then the cells were incubated with secondary antibody, Alexa Fluor 647 H+L (1:500, Invitrogen, A-21244) for 2 hours at 37°C. After the cells were washed with 1×PBS in the dark, DAPI was stained for 3 minuts as a nuclei counterstain. The cells were washed and the coverslips were mounted on glass slides with Anti-fade Mounting Medium (Beyotime). Images were captured using a confocal microscope (Leica) with a 60× oil immersion objective.

Metabolites Detection Assay

Wild type (WT) (n=6) and *Gstz1*$^{-/-}$ (n=6) adult mice (3 males and 3 females) were administered 2% (w/v) phenylalanine (Phe; Sigma, St. Louis, MO, USA) in drinking water for 1 week before sacrifice. Liver tissue (200 mg) was obtained from each mouse, and blood was washed off with physiological saline solution. The obtained liver tissues were placed into liquid nitrogen for 15 minutes and then stored at -80°C until analysis. Each 100 mg sample of liver tissue was extracted with cold acetonitrile/water (4:1, v/v) using a homogenizer. After centrifuging this mixture at 4°C and 15000 rpm for 10 minutes, the supernatant was freeze-dried at -52°C and dissolved in 100 μl acetonitrile/water (1:1, v/v). The concentrations of Phe/Tyr catabolites were detected using an Ultra High Performance Liquid Chromatography coupled to triple-guadrupole Mass Spectrometer (UHPLC-QqQ-MS).

Phenylalanine and Succinylacetone Treatment in vitro

The phenylalanine (Phe; Sigma) or succinylacetone (SA; Sigma) was added to HepG2 cell culture medium in order to investigate whether the catabolites of tyrosine metabolism were activators of KEAP1/NRF2 pathway. Phe or SA was added into the cell culture medium at a final concentration of 2.0 mM or 200 μM, respectively. The treatment time of Phe or SA was

36 hours for ARE promoter activity detection and 48 hours for western blot and chromatin immunoprecipitation assay.

Analysis of KEAP1 Modification by Mass Spectrometry

Analysis was performed using an LTQ-Orbitrap Velos (Thermo, Wilmington, DE, USA) mass spectrometer coupled with a nano-UPLC system (nanoAcquity Ultra Performance LC, Waters). Proteins were reduced with 5 mM dithiothretiol at 42°C for 30 minutes, and then alkylated with 10mM iodoacetamide in the dark at room temperature for 30 minutes. Proteins were digested with trypsin (10 ng/μl) at 37°C for 12h and resulting peptides were extracted from the gel with extracting buffer (50% acetonitrile (ACN), 5% formic acid (FA)). Peptides were separated on a self-packed capillary column (75 μm i.d. ×15cm, 3 μm C18 reverse-phase resin). Peptides were eluted using a 60 minutes nonlinear gradient: 4% B for 6 minutes, 4-10% B for 6 minutes, 10-24% B for 30 minutes, 24-34% B for 15 minutes, 34-80% B for 1 minute, 80% B for 2 minutes (Buffer A, 0.1% FA in ddH_2O; Buffer B, 0.1% FA and 98% ACN in ddH_2O; flow rate, ~300 nl/minute). The MS survey scans were acquired at a resolution of 30000, and target values of 1 000 000 ions over a mass range from 300 to 1600 m/z. Collision-induced dissociation was performed on the twenty most abundant ions per full MS scan using an isolation width of 2.0 Da. All fragmented precursor ions were actively excluded from repeated MS/MS analysis for 30 seconds. The raw files were searched with the Maxquant (v1.5.3.30) against the Swiss-Prot reviewed human database (2017-02). Full cleavage by trypsin was set including two miscleavages. Identifications satisfying the criteria, FDR<=1% at both peptide and protein levels, were considered.

RNA-sequencing (RNA-seq) Analysis

For transcriptomics profiling, Huh7 cells were infected with AdGSTZ1 (n=3) or AdGFP (n=3)

for 36 hours. Total RNA was extracted using TRIzol™ reagent (Invitrogen), and quantified using a NanoDrop ND-1000 spectrophotometer (Thermo) and a Bioanalyzer 2200 (Agilent, Santa Clara, CA, USA). High quality RNA with an RNA Integrity Number (RIN) > 8.0 was used for the construction of cDNA library. RNA-seq was performed at the Shanghai Novel Bio-Pharm Technology Co., Ltd. In brief, strand-specific RNA-seq libraries were prepared using the Total RNA-seq (H/M/R) Library Prep Kit (Vazyme Biotech, Nanjing, Jiangsu, China), and were sequenced on an Ion Torrent Proton Sequencer using the Ion PI Sequencing 200 Kit (Life Technologies, Carlsbad, CA, USA). Raw reads in FASTQ format were subjected to quality control using FastQC (http://www.bioinformatics.babraham.ac.uk/projects/fastqc/). RNA-seq reads were aligned to the reference genome using Bowtie (http://bowtie-bio.sourceforge.net). Gene expression levels were expressed as RPKM (reads per kilobase per million), and DEGs were assessed with rSeq (http://www-personal.umich.edu/~jianghui/rseq). The RNA-seq data generated in this study were deposited in the GEO database (http://www.ncbi.nlm.nih.gov/geo) under accession number GSE117822.

Immunoprecipitation (IP) Assay

The expression vector of human KEAP1 (AdKEAP1-3Flag) was made by subcloning PCR-amplified cDNA of KEAP1 into pSEB-3Flag vector (a gift from Dr. T-C He, University of Chicago, USA). The Flag-tagged KEAP1 was subcloned into the shuttle vector pAdTrack-TO4 and generated the adenoviral recombinant Ad-KEAP1-3Flag using the AdEasy system. An analogous adenovirus expressing only GFP (AdGFP) was used as control. The GSTZ1-KO HepG2 cells were seeded in two 100mm dishes and infected with AdKEAP1-3Flag, an adenovirus that expressing KEAP1 protein conjugated with Flag tag. 12 hours later, Phe was added into the cell culture medium at a final concentration of 2.0 mM.

After 48 hours, the cells were lysed for immunoprecipitation. The lysis buffer was composed of 10ml of Cell lysis buffer (50 mM Tris HCl, pH 7.4, with 150 mM NaCl, 1 mM EDTA, and 1% Triton X-100) and 1 tablet of protease inhibitor cocktail (Roche). Before use, the lysis buffer was pre-cooled on ice. Each dish of cells was rinsed twice with PBS and lysed with 1ml of lysis buffer for 30min on ice. Then the cells were scraped and the cell lysate was centrifuged at 12,000 g for 10 minutes at 4°C. The supernatant was transferred to a chilled test tube and kept on ice. The anti-Flag M2 affinity gel (Sigma, A2220) was thoroughly suspended. 40 µl of the gel was transferred to a test tube and centrifuged at 8000 g for 30 seconds at 4°C. The supernatant was discarded and the gel was washed with 0.5 ml of TBS (50 mM Tris HCl, with 150 mM NaCl, pH 7.4) twice. The gel was incubated with 1ml of cell lysate on a roller shaker at 4°C overnight. The next day, the mixture was centrifuged at 8000 g for 30 seconds at 4°C. The supernatant was removed and the gel was washed with 0.5 ml of TBS three times. Then the gel was boiled with 20 µl of 2× loading buffer for 5min and centrifuged at 8000 g for 30 seconds. The supernatants were collected and loading on SDS-PAGE for Electrophoresis. Then the gel was stained with coomassie blue and the bands indicating KEAP1 were excise for mass spectrometry to identify the modification of KEAP1.

KEAP1 Cysteine Target Validation

The Cys to Ser mutant of KEAP1 (C23, C151, C319, C406, and C513) was constructed by site-directed mutagenesis, with WT KEAP1-3Flag plasmid as a template. The sequences of mutagenesis PCR primers were presented in Appendix Table S1. The authenticity of these constructs was confirmed by DNA sequencing. The WT KEAP1, C23S, C151S, C319S, C406S or C513S mutant KEAP1 was co-transfected with Myc-tagged NRF2 plasmid into HepG2 cells, respectively. Then the expression levels of KEAP1 and NRF2 proteins were detected by western blotting.

Co-Immunoprecipitation (Co-IP) Assay

MHCC-97H cells were lysed with cell lysis buffer (Beyotime) containing a protease inhibitor cocktail (Roche) according to the manufacturer's instructions. Protein concentrations were measured using a BCA protein assay (Dingguo). Protein supernatants were incubated with Protein G Agarose Beads (Millipore) at 4°C for 1 hour, then centrifuged at 3000 rpm for 5 minutes. The supernatants were incubated with anti-NRF2 (1:20, Abcam, ab62352) or anti-SP1 (1:50, CST, #9389) antibodies overnight at 4°C, followed by incubation with Protein G Agarose Beads at 4°C for 2 hours. The beads were lightly washed twice in cell lysis buffer, followed by one wash in PBS containing 0.1% Tween-20. Immune complexes were resolved by SDS-PAGE on 10% gels, transferred to PVDF membranes, and then subjected to immunoblot analysis using the indicated antibodies.

Chromatin Immunoprecipitation (ChIP) Assay

MHCC-97H cells treated with or without Brusatol (Bru; Meilunbio, Dalian, Liaoning, China), tertiary butylhydroquinone (tBHQ; Sigma), Phe (Sigma) or SA (Sigma) were incubated with 1% formaldehyde for 10 minutes at 37˚C, followed by termination of the cross-linking reaction in 0.125 M glycine solution for 5 minutes at 37˚C. Cells were lysed with cell lysis buffer for western blotting and immunoprecipitation (Beyotime) containing 1% protease inhibitor cocktail (Roche). Precipitates were resuspended after adding ChIP Lysis Buffer (150 mM NaCl, 50 mM HEPES, 1 mM EDTA, 1% Triton X-100, 0.1% SDS, 0.1% sodium deoxycholate, pH=7.5) containing cocktail (Roche) and lysed for 30 minutes on ice. Ultrasonic pyrolysis genome using EpiSonic Multi-Functional Bioprocessor 1000 (Epigentek, Farmingdale, NY, USA) was performed to obtain 200-1000 bp DNA fragments. Supernatants were incubated with Protein G Agarose Beads (Millipore) at 4°C for 1 hour, and then centrifuged at 3000 rpm

for 1 minute. Each of supernatant was divided into four parts for Input control and incubation with antibodies. The supernatants were incubated with IgG as negative control, with anti-Histone H3 (4ug for 25 µg of chromatin, Abcam, ab12079) as positive control, or with anti-SP1 antibody (1:100, CST, #9389) overnight at 4°C, followed by incubation with Protein G Agarose Beads at 4°C for 2 hours. The beads were gently washed for 5 minutes each time in ChIP Lysis Buffer (containing 1% cocktail), high salt wash buffer (500 mM NaCl, 50 mM HEPES, 1 mM EDTA, 1% Triton X-100, 0.1% SDS, 0.1% sodium deoxycholate, pH=7.5), ChIP Lysis Buffer (containing 1% cocktail), and hiTE Buffer (50 mM Tris/HCl, 10 mM EDTA, pH=7.5). The precipitates were resuspended for 10 minutes in ChIP Elution Buffer (50 mM Tris/HCl, 10 mM EDTA, 1% SDS). Following elution, immunoprecipitated DNA was extracted and subjected to PCR analysis. The 20 µl PCR reaction system was assembled as follows: 2 µl DNA, 0.5 µl forward primer (20 µM), 0.5 µl reverse primer (20 µM), 10 µl 2×Taq PCR Green Mix (Dingguo, Beijing, China), and 7 µl nuclease-free water. The reaction conditions were: one cycle at 96°C for 2 minutes, followed by 35 cycles of amplification at 96°C for 20 seconds, then at 55°C for 30 seconds, and then at 72°C for 30 seconds. All samples were run in triplicate in each experiment. The sequences of the primers for human IGF1R promoter were showed in Appendix Table S1.

Flow Cytometry-Based Detection of Apoptosis

The apoptosis of HCC cells was evaluated by flow cytometry using an Annexin V-Fluorescein isothiocyanate/propidium iodide (V-FITC/PI) kit (Beyotime) according to the manufacturer’s instructions. Cells were seeded into 6-well plates with three replicate wells for each group. PPP treatment was introduced after the cells were adhered. The cells were then collected, rinsed in pro-cooling PBS twice, and then centrifuged at 1000 g for 5 minutes. The supernatant was removed, and the cells were resuspended after in 100 µl binding buffer.

Next, cells were stained with 5 µl Annexin V-FITC and 10 µl PI for 15 minutes in the dark, followed by the addition of 400 µl binding buffer. Cells in early stages of apoptosis were stained with Annexin V+/PI-.The rate of apoptosis was analyzed by FACSCalibur™ Flow Cytometer (BD Biosciences, Franklin, NJ, USA).

Enzyme-Linked Immunosorbent Assay (ELISA)

Whole blood samples, obtained from the mouse orbit, were incubated at 37°C for 2 hours, followed by centrifugation at 1000 g for 20 minutes. The separated sera were collected. Serum levels of alanine aminotransferase (ALT) and alpha-fetoprotein (AFP) were assessed using a Mouse ALT and AFP ELISA Kit (KaLang, Shanghai, China) according to the manufacturer's protocol. Serum samples or standards (50 µl/well) were added to a coated 96-well ELISA microplate. After incubation at 37°C for 30 minutes, the wells were washed five times with 1X Wash Buffer. Then, 50 µl anti-ALT or AFP antibody conjugated with HRP was added to the wells and allowed to incubate at 37°C for 30 minutes. After washing five times with 1X Wash Buffer, TMB Substrate (50 µl/well) was added into the wells with and allowed to incubate for 10 minutes at 37°C in the dark. Next, stop buffer (50 µl/well) was added into the wells for induction of the blue to yellow color change. The optical density values (ODs) at 450 nm were recorded with Synergy™ H1 Hybrid multimode microplate reader (BioTek, Winooski, VT, USA). The standard curve was plotted using GraphPad prism 7 software, and the ALT and AFP values were calculated using the standard curve equation.

Luo J, Deng ZL, Luo X, Tang N, Song WX, Chen J, et al. A protocol for rapid generation of recombinant adenoviruses using the AdEasy system. Nat Protoc 2007; 2: 1236-1247.

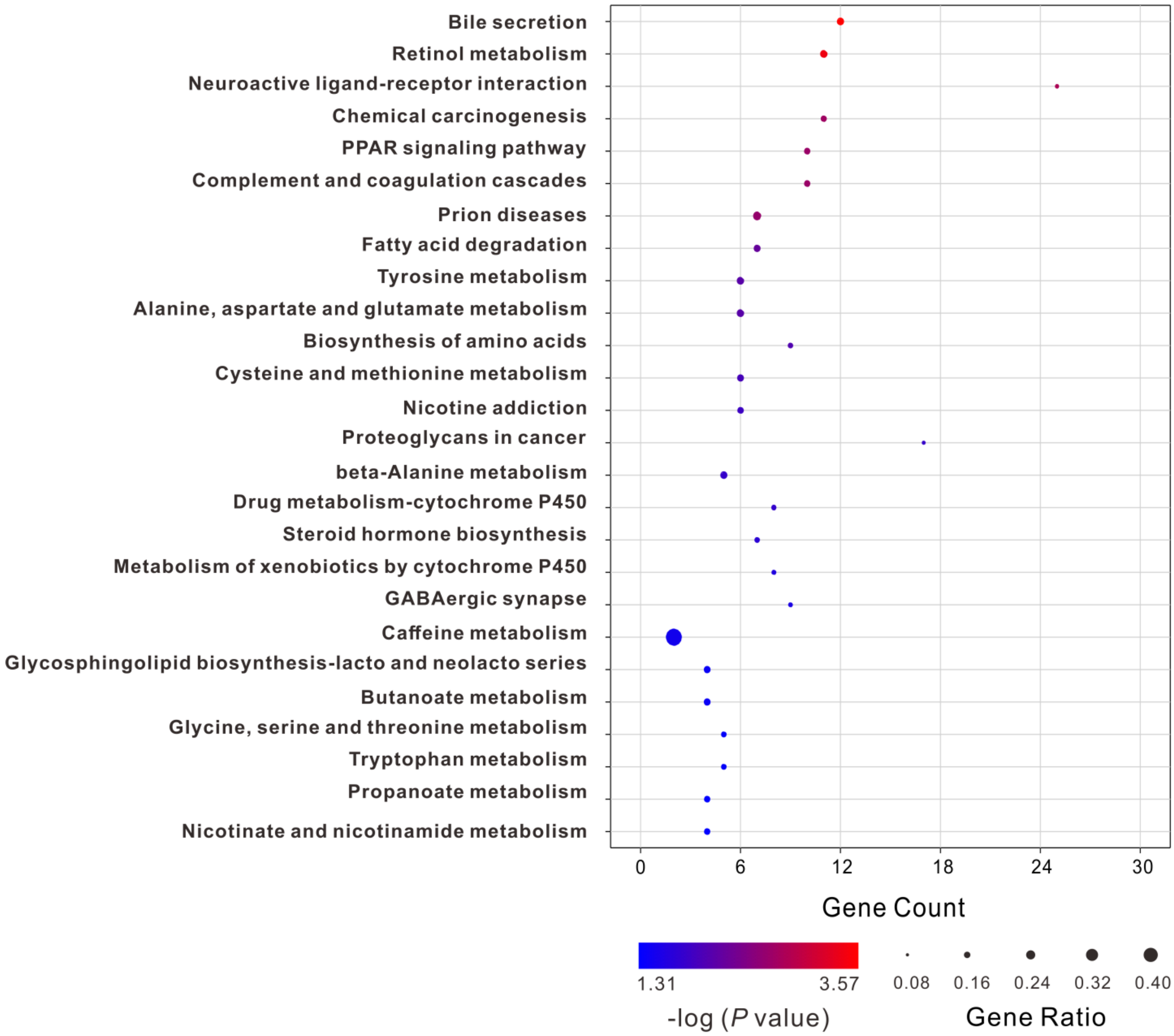

Appendix Figure S1. Significantly enriched Kyoto Encyclopedia of Genes and Genomes (KEGG) pathways associated with differentially expressed genes (DEGs). The dot size indicated the ratio of DEGs which participate in pathways, and the gradient colors from red to blue indicated ascending enrichment *P*-values.

Appendix Table S1. Primer sequences used in the study

Name	Sequences
**Primers for quantitative RT-PCR**	
ACTIN	Forward: AGGCCAACCGCGAGAAGATGACC
	Reverse: GAAGTCCAGGGCGACGTAGCAC
GSTZ1	Forward: CAGAGCACAGCGGGCATATA
	Reverse: GCTCAGTGGGTGTATCTGGC
IGF1R	Forward: ATGACATTCCTGGGCCAGTG
	Reverse: TAGCTTGGCCCCTCCATACT
NQO1	Forward: CCAGCAGACGCCCGAAT
	Reverse: CCAAGTGATGGCCCACAGA
MRP2	Forward: ACGGGCACATCACCATCAAG
	Reverse: CTCCAGGCAGCATTTCCAAG
GCLM	Forward: GACAAAACACAGTTGGAACAGC
	Reverse: CAGTCAAATCTGGTGGCATC
TXNRD1	Forward: CCCTTTCTTTCATTCCCTCTCTG
	Reverse: ATTCACCCACCTTTTGTCCCT
G6PD	Forward: TGACCTGGCCAAGAAGAAGA
	Reverse: CAAAGAAGTCCTCCAGCTTG
HO1	Forward: AACTTTCAGAAGGGCCAGGT
	Reverse: CTGGGCTCTCCTTGTTGC
IRS1	Forward: AAGTGGCGGCACAAGTCG
	Reverse: ACGTCACCGTAGCTCAAGTCC
IRS2	Forward: TGTACCGTCTGTGCCTGTCTG
	Reverse: TGGTCTCGTGGATGTTCTGC

PIK3CA	Forward:	TGCTGTTCGGTGCTTGGA
	Reverse:	CTCTGGCTAACTGTTTTATTGTGC
PDPK1	Forward:	CTGTATGACGCCGTGCCC
	Reverse:	CCAAGGATTTTCCCAAACTTGA
BRAF	Forward:	GGCACGGAGCAACCCCA
	Reverse:	CAGGAAATATCAGTGTCCCAACC
RPS6KA1	Forward:	GATCGGCAGTGGGAAGTTTAC
	Reverse:	TGGGCTTGGAGCTGTTGAGT
BCL2	Forward:	GGTGAACTGGGGGAGGATTG
	Reverse:	GGCCAAACTGAGCAGAGTCT
BCLG	Forward:	CCATTGCCAACCGAGTAGC
	Reverse:	GGCCCATCAAAGCCTTATCT
BAX	Forward:	CATGAAGACAGGGGCCCTTT
	Reverse:	GGAAAAAGACCTCTCGGGGG
CASP9	Forward:	TGCTGCGTGGTGGTCATT
	Reverse:	CTGGCTCGGGGTTACTGC
CASP7	Forward:	GTCCTCGTTTGTACCGTCCC
	Reverse:	TCGGCATCTTTGTCTGTTCC
NFKB1	Forward:	GATTTCGTTTCCGTTATGTATGTG
	Reverse:	TGTCCTTGGGTCCAGCAGTT
BIRC3	Forward:	CTTTTGCTGTGATGGTGGACTC
	Reverse:	CTCCTGGGCTGTCTGATGTG

Primers for GSTZ1 sgRNA

GSTZ1 sg1	Forward:	CACCGCACGCTGGCCCTCTTCTTT
	Reverse:	AAACAAAGAAGAGGGCCAGCGTGC

GSTZ1 sg2	Forward:	CACCGCTGGATGCCACCAGCGATG
	Reverse:	AAACCATCGCTGGTGGCATCCAGC
GSTZ1 sg3	Forward:	CACCGCCCAGAACGCCATCACTTG
	Reverse:	AAACCAAGTGATGGCGTTCTGGGC

Primers for TA-clone

GSTZ1-seq1 and 2	Forward:	TGGATGGCTGACTAGGGTAG
	Reverse:	GCTGCTCTGGTTAAAGGCA
GSTZ1-seq3	Forward:	GGACCATGCAAGGGAGAA
	Reverse:	TTAAGACGGTTTAGTGGGAGTG

Primers for wild type and mutant KEAP1

KEAP1	Forward:	CGGGGTACCATGGGCCAGCCAGATCCCAGGCCTAGC
	Reverse:	CCCAAGCTTTTAAATCTTATCGTCGTCATCCT
KEAP1	Forward:	TGCCCCTGCAGTCACAGAGCCCTGAGGG
C23S	Reverse:	CCCTCAGGGCTCTGTGACTGCAGGGGCA
KEAP1	Forward:	CGCAGGTGATGCCCAGCCGGGCGCC
C319S	Reverse:	GGCGCCCGGCTGGGCATCACCTGCG
KEAP1	Forward:	ATCAGTGGTCGCCCAGCGCCCCCATGA
C406S	Reverse:	TCATGGGGGCGCTGGGCGACCACTGAT
KEAP1	Forward:	AGCGGGGCAGGCGTCAGCGTCCTGCAC
C513S	Reverse:	GTGCAGGACGCTGACGCCTGCCCCGCT
GSTZ1	Forward:	CGCGGATCCACCATGGGCCAGGCGGGGAAGCCCATCCTCTAT
	Reverse:	CCCAAGCTTCTAGGCCCTCAGCTCAGTGGGTGT

Primers for IGF1R promoter construct

IGF1R promoter	Forward:	CTAGCTAGCCACCAGGCGAACTCGAGA
	Reverse:	CCGCTCGAGGCGAAGGAAACAATACTCCGAA

**Primers for ChIP in the IGF1R promoter**	
IGF1R promoter	Forward: GGCTGGGGCTCTTGTTTAC
	Reverse: TCGAGTTCGCCTGGTGC
**Primers for IGF1R shRNA**	
IGF1R sh1	Forward: TGCGGTAGTTGTACTCATTGTTCAAGAGACAATGAGTACAACTACCGCTTTTTTC
	Reverse: TCGAGAAAAAAGCGGTAGTTGTACTCATTGTCTCTTGAACAATGAGTACAACTACCGCA
IGF1R sh2	Forward: TGGCCAGAAATGGAGAATAATTCAAGAGATTATTCTCCATTTCTGGCCTTTTTTC
	Reverse: TCGAGAAAAAAGGCCAGAAATGGAGAATAATCTCTTGAATTATTCTCCATTTCTGGCCA
IGF1R sh3	Forward: TGGATATTGGGCTTTACAACTTCAAGAGAGTTGTAAAGCCCAATATCCTTTTTTC
	Reverse: TCGAGAAAAAAGGATATTGGGCTTTACAACTCTCTTGAAGTTGTAAAGCCCAATATCCA

Appendix Table S2. The characteristics of antibodies

Protein	Application	Origin	Dilution
GSTZ1-1	WB&IHC	GTX106109; GeneTex, Irvine, CA, USA	1000&100
IGF1Rβ	WB	#9750; CST, Beverly, MA, USA	1000
pIGF1Rβ (Tyr1131)	WB	#3021; CST, Beverly, MA, USA	1000
NQO1	WB	BS6833; Bioworld, Louis, MN, USA	1000
TXNRD1	WB	GTX108727; GeneTex, Irvine, CA, USA	1000
HO1	WB	GTX101147; GeneTex, Irvine, CA, USA	1000
NRF2	WB&IF&Co-IP	ab62352; Abcam, Cambridge, MA, USA	1000&300&20
Myc	WB	#2278; CST, Beverly, MA, USA	1000
Flag	WB	MA1-91878; Thermo, Waltham, MA, USA	500
AKT	WB	BS1810; Bioworld, Louis, MN, USA	1000
pAKT (Thr308)	WB	BS4009; Bioworld, Louis, MN, USA	1000
ERK	WB	#4695; CST, Beverly, MA, USA	1000
pERK (Thr202/204)	WB	#4370; CST, Beverly, MA, USA	2000
pBAD (Ser136)	WB	#4366; CST, Beverly, MA, USA	1000
pCREB (Ser133)	WB	RLP0075; Ruiying, Suzhou, Jiangsu, China	1000
BCL2	WB	RLM3041; Ruiying, Suzhou, Jiangsu, China	1000
Caspase9	WB	RLM3077; Ruiying, Suzhou, Jiangsu, China	1000
Caspase3	WB	#9665; CST, Beverly, MA, USA	1000
actin	WB	BL005B; Biosharp, Hefei, Anhui, China	1000
tubulin	WB	66240-1-Ig; Proteintech, Suite, IL, USA	10000
laminB1	WB	AP6001; Bioworld, Louis, MN, USA	5000
Goat anti-Rabbit IgG	WB	ab6721; Abcam, Cambridge, MA, USA	10000
Goat anti-Mouse IgG	WB	ab6789; Abcam, Cambridge, MA, USA	5000

IGF1Rβ	IHC	#3027; CST, Beverly, MA, USA	100
pIGF1Rβ (Tyr1161)	IHC	ab39398; Abcam, Cambridge, MA, USA	50
Alexa Fluor 647 H+L	IF	A-21244; Invitrogen, Carlsbad, CA, USA	500
Flag M2 Affinity Gel	IP	A2220; Sigma, St. Louis, MO, USA	40 µl
SP1	Co-IP&ChIP	#9389; CST, Beverly, MA, USA	50&100
Histone H3	ChIP	ab12079; Abcam, Cambridge, MA, USA	4:25 µg

Appendix Table S3. Other resources table

**Cell Lines**	
HEK293T	Lab stock
Hep3B	Lab stock
HepG2	ATCC, Cat#HB-8065
Huh7	Lab stock
MHCC97H	Lab stock
MIHA	Lab stock
PLC/PRF/5	Lab stock
SK-Hep1	Lab stock
**Recombinant DNA**	
LentiCrisprv2	Lab stock
pAdTrack-TO4	Dr. T-C He, University of Chicago, USA
pcDNA-5Myc	Prof. Wei LV, Shanghai Institute of Nutrition and Health, China
pGL3-ARE	Prof. Yiguo Zhang, Chongqing University, China
pGL3-IGFIR promoter	Cat#A80241/Y0009053-1, Tsingke Biological Technology, Beijing, China
pLL3.7	kindly provided by Prof. Bing Sun, Shanghai Institute of Biochemistry and Cell Biology, Chinese Academy of Sciences, China
pLL3.7-shIGFIR	This study
pOTB7-GSTZ1	GeneCopoeia, Cat#FL09522
pRL-TK	Lab stock
pSEB-3Flag	Dr. T-C He, University of Chicago, USA

**Bacterial and Virus Strains**	
AdEasy-BJ5183 E. coli	Lab stock
AdGSTZ1	This study
AdKeap1-3Flag	This study
DH10B Chemically Competent E. coli	Lab stock
Lenticrispr-GSTZ1	This study
**Animal Strains**	
BALB/c nude mice (male)	Experimental Animal Center of Chongqing Medical University
129-GSTZ1^{tm1Jmfc}/Cnbc	EMMA: CNB-CSIC, Cat#B001301; RRID: IMSR_EM:04481
**Chemicals and Recombinant Proteins**	
NTBC	N843653, Macklin, Shanghai, China
2×Taq PCR Green Mix	PER 007, Dingguo, Beijing, China
Anti-fade Mounting Medium	P0126, Beyotime, Shanghai, China
Brusatol (Bru)	MB7292, Meilunbio, Dalian, Liaoning, China
Carbon tetrachloride (CCl_4)	C805332, Macklin, Shanghai, China
Cell lysis buffer for Western and IP	P0013, Beyotime, Shanghai, China
DAPI	10236276001, Roche, Mannheim, Germany
Diethylnitrosamine (DEN)	N0756, Sigma, St. Louis, MO, USA
DMEM	SH30243.01, Hyclone, Waltham, MA, USA
Lipofectamine 2000	11668019, Invitrogen, Carlsbad, CA, USA
MEM	SH30024.01, Hyclone, Waltham, MA, USA
N-acetylcysteine (NAC)	Cat# S0077, Beyotime, Shanghai, China
Opti-MEM	31985070, Gibco, Carlsbad, CA, USA

Penicillin-Streptomycin Solution	SV30010, Hyclone, Waltham, MA, USA
Phenylalanine (Phe)	P5482, Sigma, St. Louis, MO, USA
Picropodophyllin (PPP)	477-47-4, MCE, Monmouth, NJ, USA
Polybrene	40804ES76, Yeasen, Shanghai, China
Recombinant Human IGF-I	P5502, Beyotime, Shanghai, China
RIPA Lysis Buffer (Strong)	CW2333S, Cwbiotech, Beijing, China
Succinylacetone (SA)	D1415, Sigma, St. Louis, MO, USA
Suero fetal bovino esteril	Natocor, Villa Carlos Paz, Córdoba, Argentina
Tertiary butylhydroquinone (tBHQ)	112941, Sigma, St. Louis, MO, USA
TRIzol™ Reagent	15596026, Invitrogen, Carlsbad, CA, USA
Trypsin	15050057, Gibco, Carlsbad, CA, USA
Trypsin-EDTA	25200072, Gibco, Carlsbad, CA, USA
**Critical Commercial Assays**	
Annexin V-FITC Apoptosis Detection Kit	C1062L, Beyotime, Shanghai, China
BCA protein assay Kit	BCA02, Dingguo, Beijing, China
Cell-Light™ EdU Apollo®643 Kit	C10310-2, RiboBio, Guangzhou, Guangdong, China
EDTA-free Protease Inhibitor Cocktail Tablets	04693132001, Roche, Mannheim, Germany
DAB kit	ZLI-9019, ZSGB-BIO, Beijing, China
Dual-Luciferase® Reporter Assay System	E1910, Promega, Madison, WI, USA
Elivision™ plus Polyer HRP IHC Kit	KIT-9901, Maxim, Fuzhou, Fujian, China
Genomic DNA Purification Kit	GP0155, Genloci, Nanjing, Jiangsu, China
iTaq™ Universal SYBR® Green Supermix	1725121, Bio-Rad, Hercules, CA, USA
Mouse AFP ELISA Kit	KL-AFP-Mu, KaLang, Shanghai, China

Mouse ALT ELISA Kit	KL-ALT-Mu, KaLang, Shanghai, China
Nuclear and Cytoplasmic Protein Extraction Kit	P0028, Beyotime, Shanghai, China
PrimeScript® RT reagent Kit	RR047A, Takara, Shiga, Japan
**Softwares**	
GraphPad Prism 7.0	GraphPad Software, https://www.graphpad.com/scientific-software/prism
Image J 2.0	Rawak Software, https://imagej.nih.gov/ij/
Image-pro plus 7.0	Media Cybernetics, http://www.mediacy.com/imageproplus
Maxquant (v1.5.3.30)	http://www.maxquant.org/
