# Supplementary material for: GSTZ1‐1 Deficiency Activates NRF2/IGF1R Axis in HCC via Accumulation of Oncometabolite Succinylacetone
Source: EMBO J. 2019 Jun 28;38(15):e101964. doi: 10.15252/embj.2019101964 (PMC6669923; doi:10.15252/embj.2019101964)
Supplement: Supplementary file 2 — Expanded View Figures PDF [file EMBJ-38-e101964-s002.pdf]

# Expanded View Figures

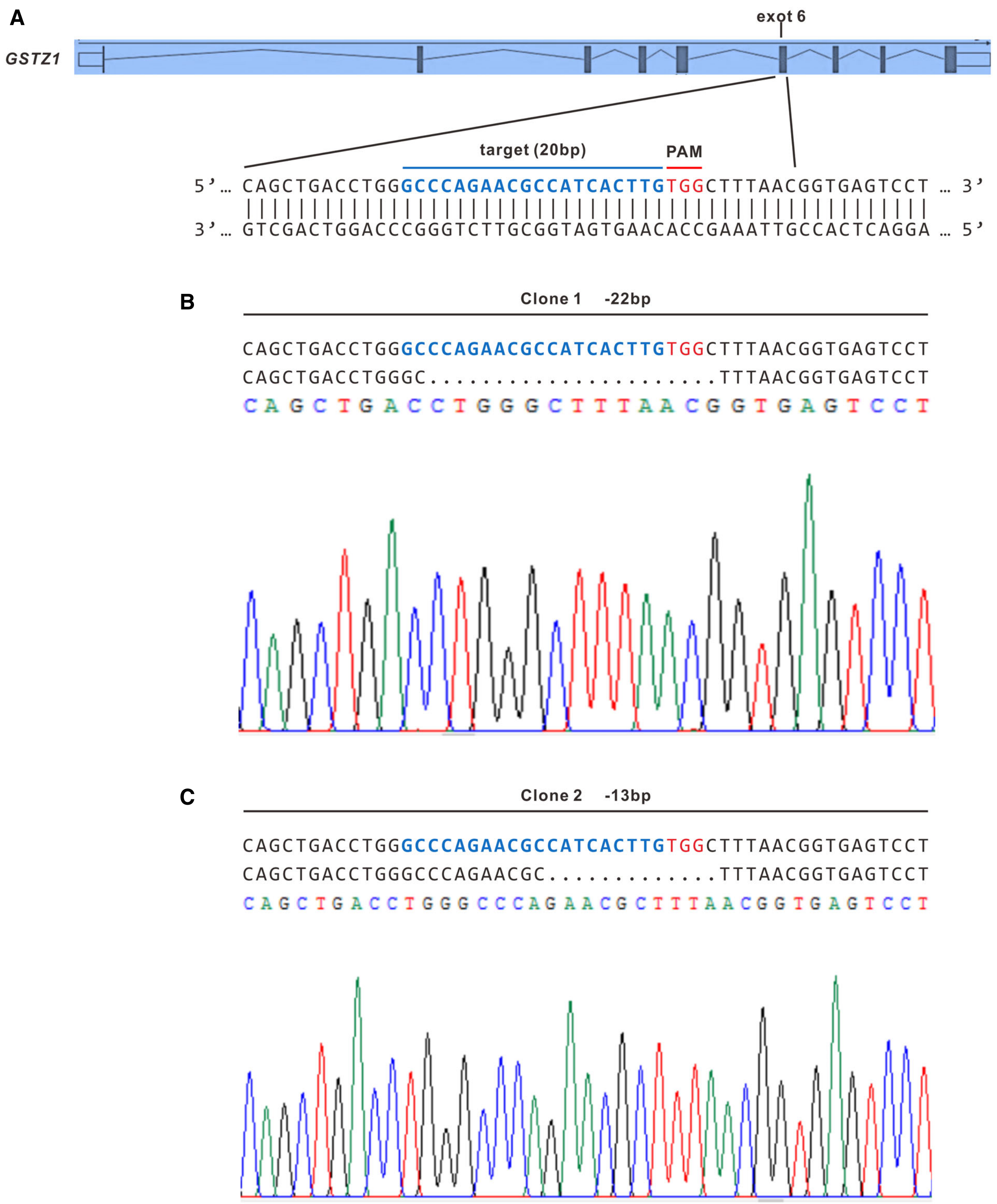


**Figure EV1. Knockout of *GSTZ1* in HepG2 cell line using the CRISPR/Cas9 system.**

A Schematic representation of *GSTZ1* locus with the targeting sequence (blue) of small guide RNA (sgRNA) and the protospacer adjacent motif (PAM) (red).

B, C Indel mutations of *GSTZ1* from two single-cell clones identified by sequencing. Dashes represent deleted bases.

© 2019 The Authors


© 2019 The Authors

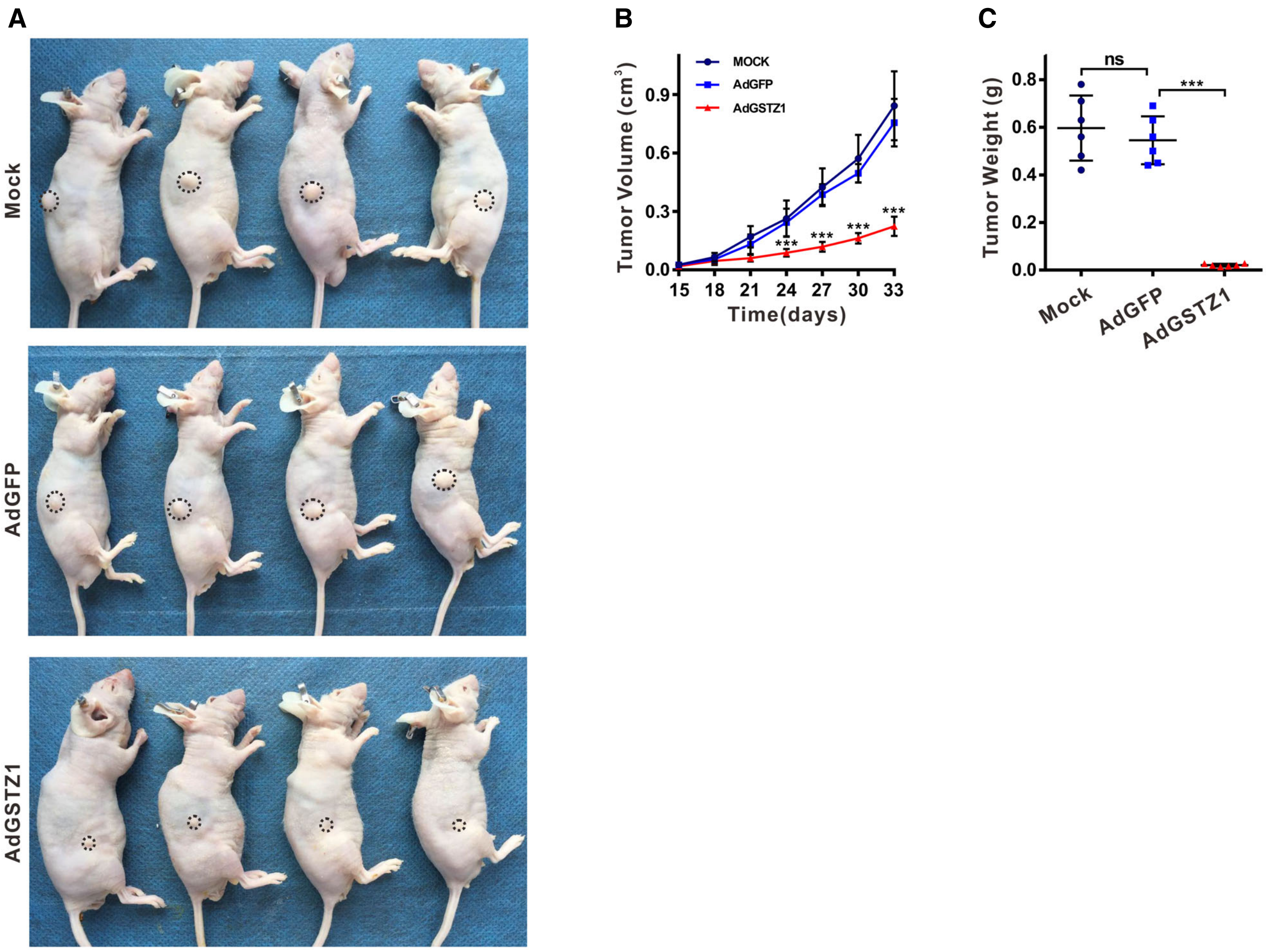


**Figure EV2. GSTZ1-1 suppresses tumor growth in the xenograft implantation model.**

MHCC-97H cells were infected with adenoviruses expressing GSTZ1-1 (AdGSTZ1) or green fluorescent protein (AdGFP) for 36 h. The infected cells ($1 \times 10^6$ cells/injection) were injected subcutaneously into the flanks of male nude mice ($n = 6$, each group), which were sacrificed on day 33.

A Representative images of tumor-bearing nude mice. The dotted circles indicate the location and mass of tumor implants.
B Tumor growth curves.
C Tumor weights assessed after sacrifice.

Data information: Values are shown as means ± SD ($n = 6$), ***$P < 0.001$, one-way ANOVA followed by the Tukey test. Abbreviations: ns, not significant.

 © 2019 The Authors

 © 2019 The Authors

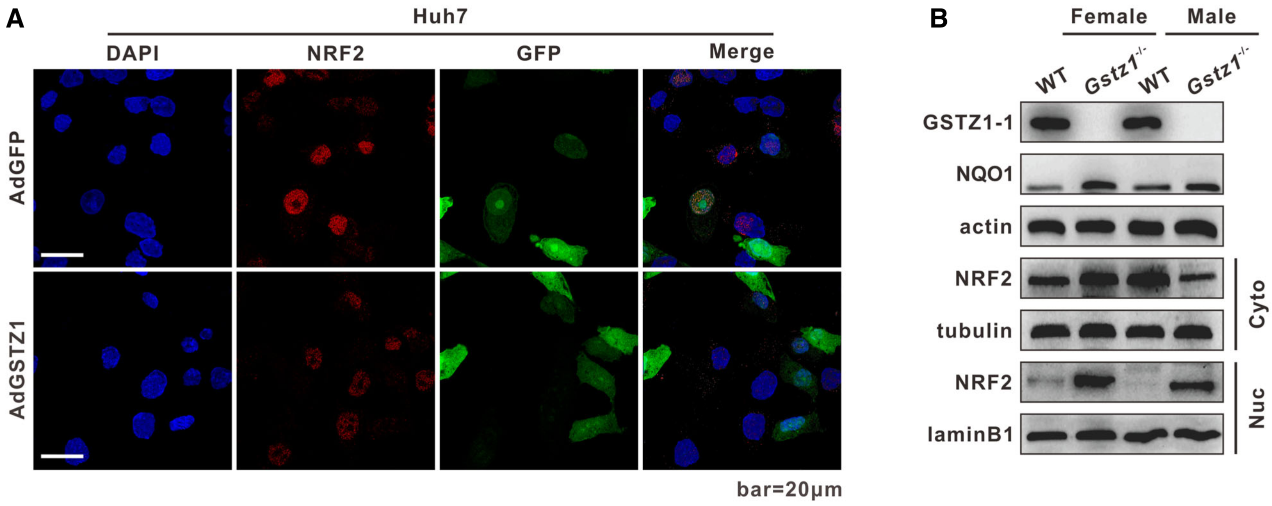


**Figure EV3. GSTZ1-1 negatively regulates NRF2 nuclear translocation.**

A Representative images of NRF2 immunofluorescence in GSTZ1-1-overexpressing Huh7 cells. Scale bars: 20 μm.
B Western blotting shows NQO1 expression and cytoplasmic and nuclear expression of NRF2 in liver tissues from wild-type (WT) and *Gstz1*$^{-/-}$ mice.

Data information: Abbreviations: DAPI, 4′,6-diamidino-2-phenylindole; NRF2, nuclear factor erythroid 2-related factor 2; GFP, green fluorescent protein; WT, wild type; NQO1, NAD(P):H quinone oxidoreductase 1.

© 2019 The Authors

© 2019 The Authors

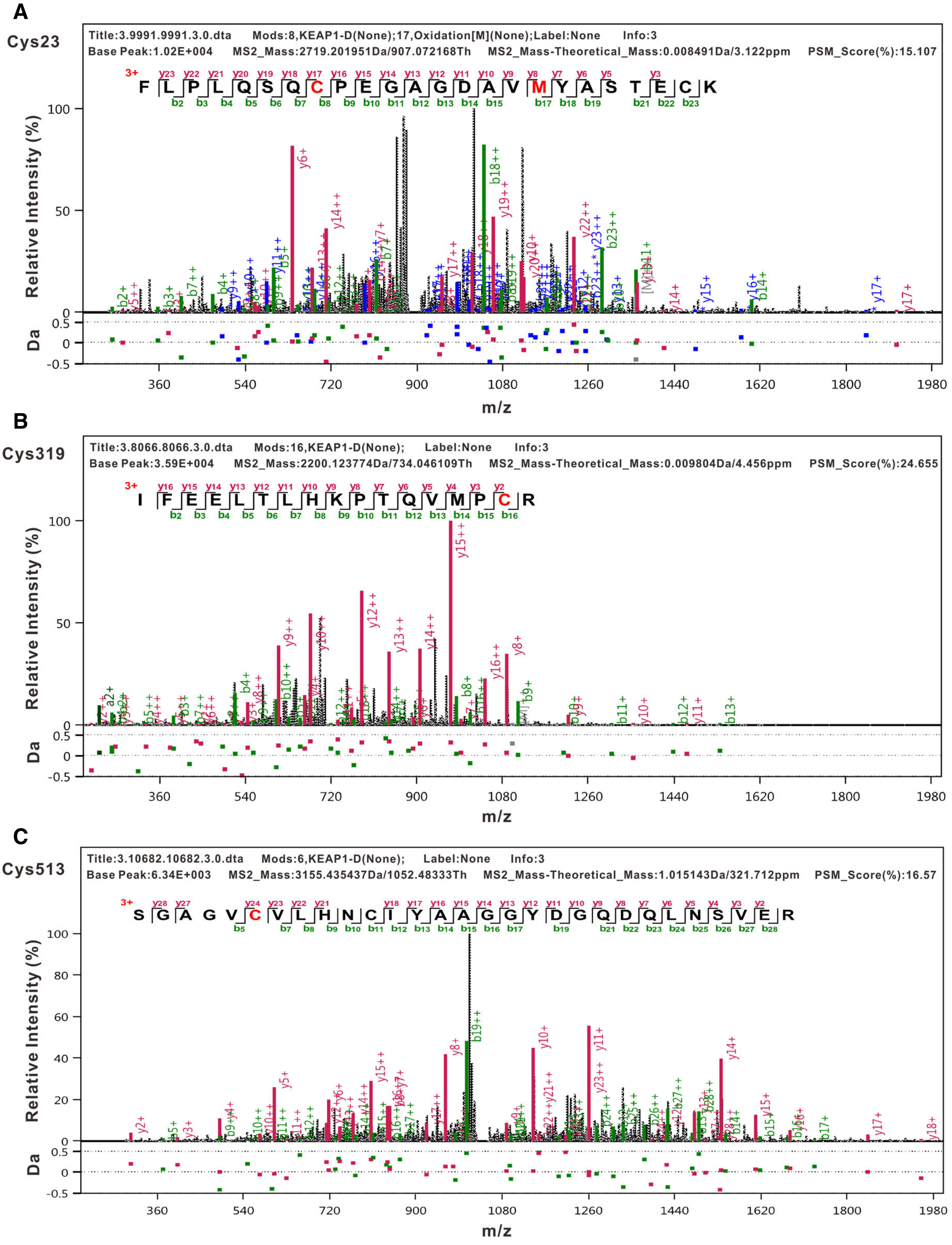


**Figure EV4. Succinylacetone causes alkylation of human KEAP1 cysteine residues.**

A–C Tandem mass spectrometry (UPLC-MS/MS) analysis of immunoprecipitated kelch-like ECH-associated protein 1 (KEAP1) identified succinylacetone (SA) modification at residues Cys23 (A), Cys319 (B), and Cys513 (C).

 © 2019 The Authors

 © 2019 The Authors

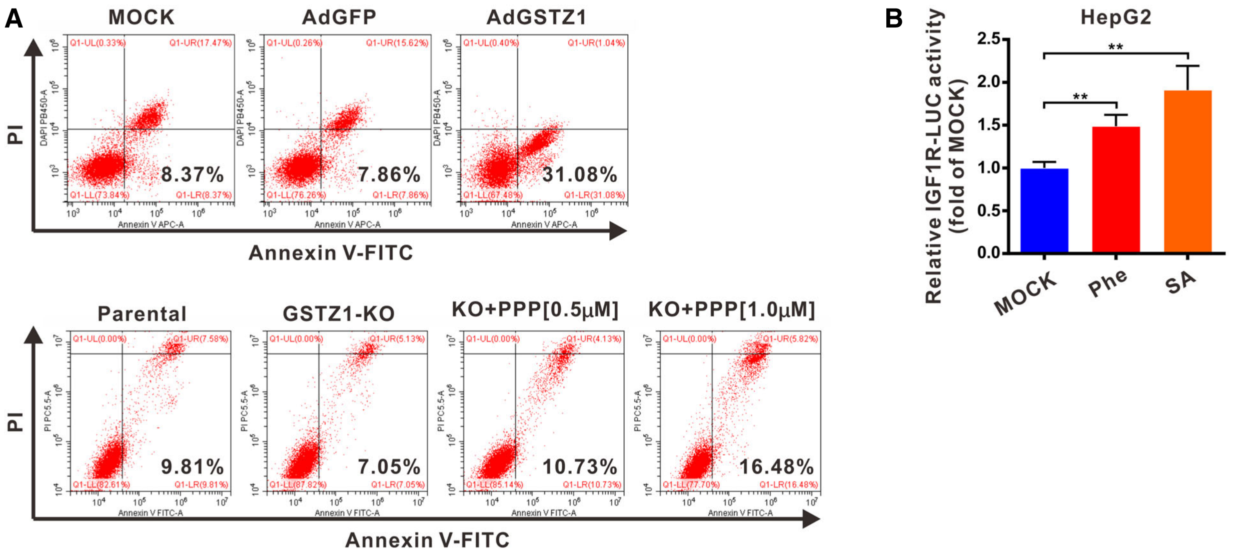


**Figure EV5. Apoptosis in hepatoma cell lines and luciferase activity of IGF1R promoter in HepG2 cells treated with metabolites.**

A Flow cytometry detection of apoptosis, conducted using Annexin V–FITC/PI double staining in GSTZ1-1-overexpressing (GSTZ1-OE) Huh7 (top) and *GSTZ1* knockout (GSTZ1-KO) HepG2 (bottom) cells. GSTZ1-KO cells were treated with or without different doses of PPP (0.5 μM and 1.0 μM) for 24 h.

B Luciferase activity of human *IGF1R* promoter in HepG2 cells treated with Phe (2.0 mM) and SA (200 μM) for 36 h. Values are shown as means ± SD ($n$ = 3), **$P < 0.01$, one-way ANOVA followed by the Tukey test.

Data information: Abbreviations: Phe, phenylalanine; SA, succinylacetone; AdGFP, adenoviruses expressing GFP; AdGSTZ1, adenoviruses expressing GSTZ1-1; PPP, picropodophyllin.


© 2019 The Authors                                The EMBO Journal e101964 | 2019
