# Supplementary material for: GSTZ1‐1 Deficiency Activates NRF2/IGF1R Axis in HCC via Accumulation of Oncometabolite Succinylacetone
Source: EMBO J. 2019 Jun 28;38(15):e101964. doi: 10.15252/embj.2019101964 (PMC6669923; doi:10.15252/embj.2019101964)
Supplement: Supplementary file 3 — Table EV1 [file EMBJ-38-e101964-s003.docx]

**Table EV1.** Candidate differentially expressed genes between paired carcinoma and para-carcinoma tissue in EpCAM positive and EpCAM negative HCC.

| **Gene Symbol** | **Gene ID** | **GenBank ID** | **logFC in EpCAM^+^ HCC** | **logFC in EpCAM^-^ HCC** | **Up or down- regulated** |
| --- | --- | --- | --- | --- | --- |
| *ACADL* | 33 | NM_001608 | -2.2138 | -1.3454 | down |
| *PCK1* | 5105 | NM_002591 | -3.5980 | -2.7977 | down |
| *GLYAT* | 10249 | NM_201648 | -2.7098 | -1.7124 | down |
| *GSTZ1* | 2954 | NM_001513 | -2.5621 | -1.9245 | down |
| *PDX1* | 3651 | NM_000209 | -2.1482 | -2.2681 | up |
| *GLYATL1* | 92292 | NM_080661 | -3.0013 | -1.9236 | down |
| *NEUROD1* | 4760 | NM_002500 | -3.0877 | -2.8111 | down |
| *PDGFRA* | 5156 | NM_006206 | -2.1938 | -2.8404 | up |
| *ACSM2B* | 348158 | NM_001105069 | -2.3177 | -1.2885 | down |
| *SHB* | 6461 | NM_003028 | -2.0189 | -1.7985 | down |
| *SLC27A5* | 10998 | NM_012254 | -2.7452 | -1.9444 | down |
| *SULT1B1* | 27284 | NM_014465 | -2.4801 | -2.0566 | down |
| *IAPP* | 3375 | NM_000415 | -2.5015 | -2.0922 | down |
| *FABP5* | 2171 | NM_001444 | 2.0559 | 1.3921 | up |
| *FGF10* | 2255 | NM_004465 | -2.0328 | -1.8564 | down |
| *PDGFRA* | 5156 | NM_006206 | -2.3303 | -2.1714 | down |
| *FABP7* | 2173 | NM_001446 | -2.1992 | -1.9190 | down |
| *REPS2* | 9185 | NM_004726 | -2.1438 | -1.9239 | down |
