# Supplementary material for: GSTZ1‐1 Deficiency Activates NRF2/IGF1R Axis in HCC via Accumulation of Oncometabolite Succinylacetone
Source: EMBO J. 2019 Jun 28;38(15):e101964. doi: 10.15252/embj.2019101964 (PMC6669923; doi:10.15252/embj.2019101964)
Supplement: Supplementary file 4 — Table EV2 [file EMBJ-38-e101964-s004.docx]

**Table EV2.** Cysteine residues in KEAP1 found to be modified in *GSTZ1* knockout cells by mass spectrometry.

| **SA Modified Residue** | **Peptide Amino Acid Position** | **Peptide Sequence** | **Score** |
| --- | --- | --- | --- |
| 23 | 16-39 | FLPLQSQCPEGAGDAVMYASTECK | 56 |
| 319 | 304-320 | IFEELTLHKPTQVMPCR | 97 |
| 406 | 381-413 | NNSPDGNTDSSALDCYNPMTNQWSPCAPMSVPR | 44 |
| 513 | 508-536 | SGAGVCVLHNCIYAAGGYDGQDQLNSVER | 87 |

Abbreviations: SA, succinylacetone.
