# Supplementary material for: GSTZ1‐1 Deficiency Activates NRF2/IGF1R Axis in HCC via Accumulation of Oncometabolite Succinylacetone
Source: EMBO J. 2019 Jun 28;38(15):e101964. doi: 10.15252/embj.2019101964 (PMC6669923; doi:10.15252/embj.2019101964)

Fig.1E

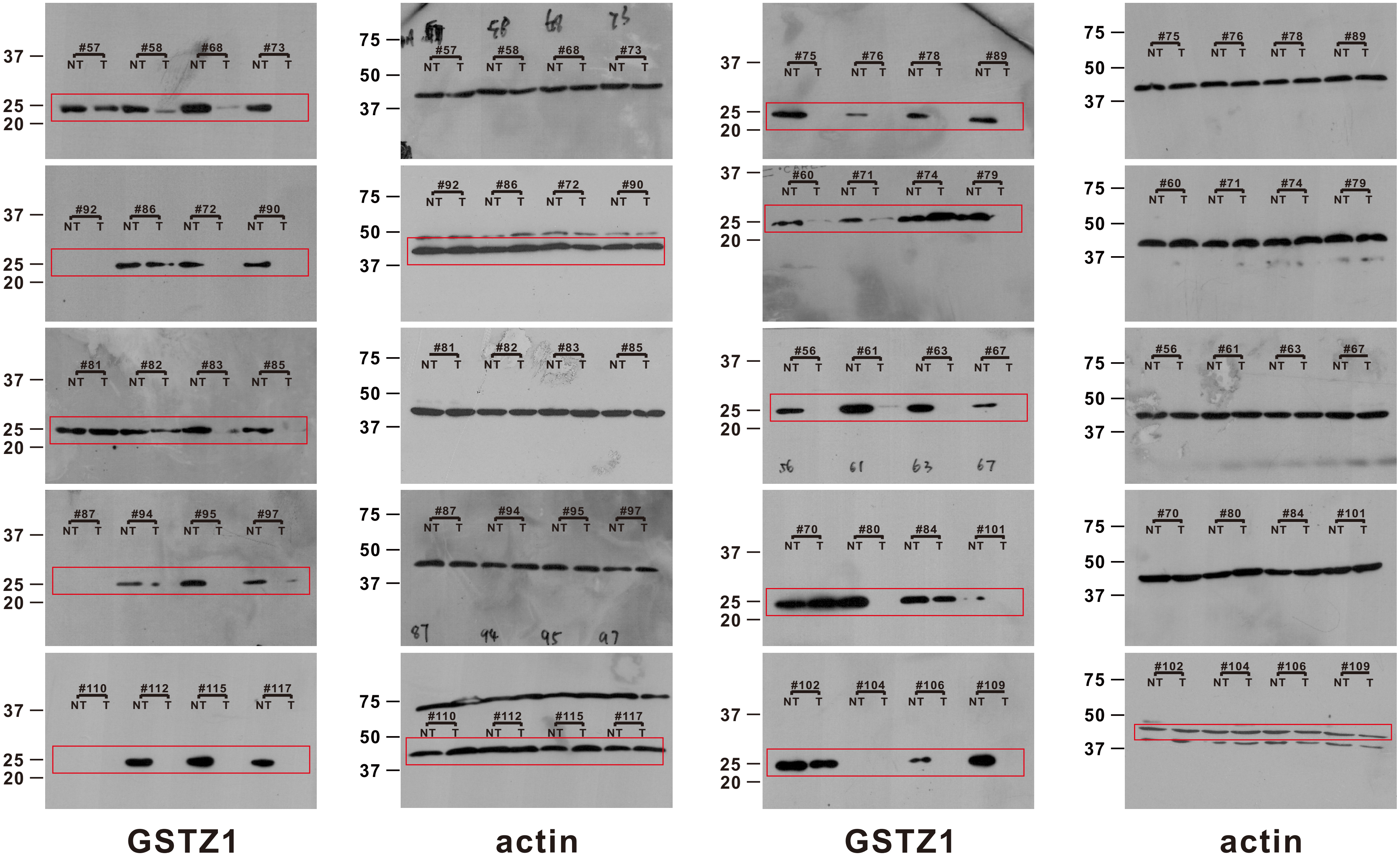

Supplement: Supplementary file 6 — Source Data for Figure 1 [file EMBJ-38-e101964-s005.zip › embj2019101964-sup-0005-SDataFig1/SourceDataGelsFig1.pdf]
