# Supplementary figures and images for: GSTZ1‐1 Deficiency Activates NRF2/IGF1R Axis in HCC via Accumulation of Oncometabolite Succinylacetone
Source: EMBO J. 2019 Jun 28;38(15):e101964. doi: 10.15252/embj.2019101964 (PMC6669923; doi:10.15252/embj.2019101964)

**Fig.2A**

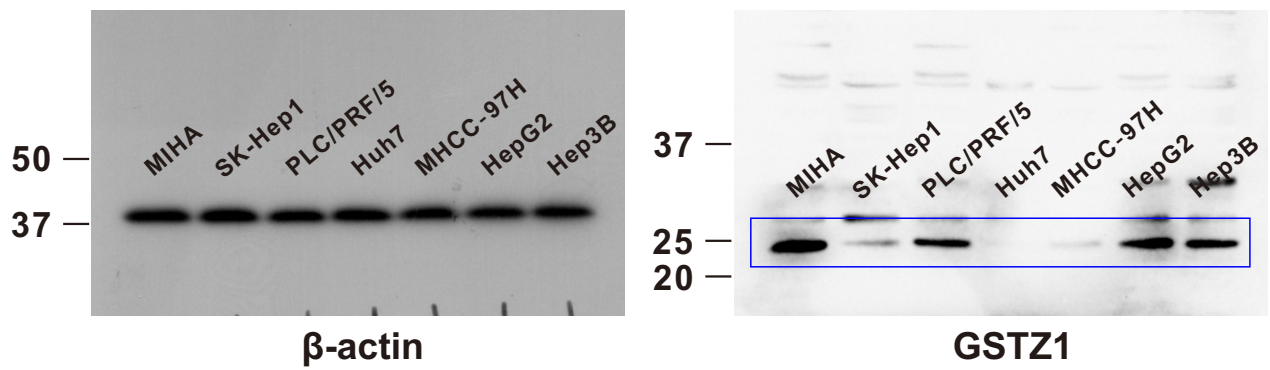

**Fig.2B**

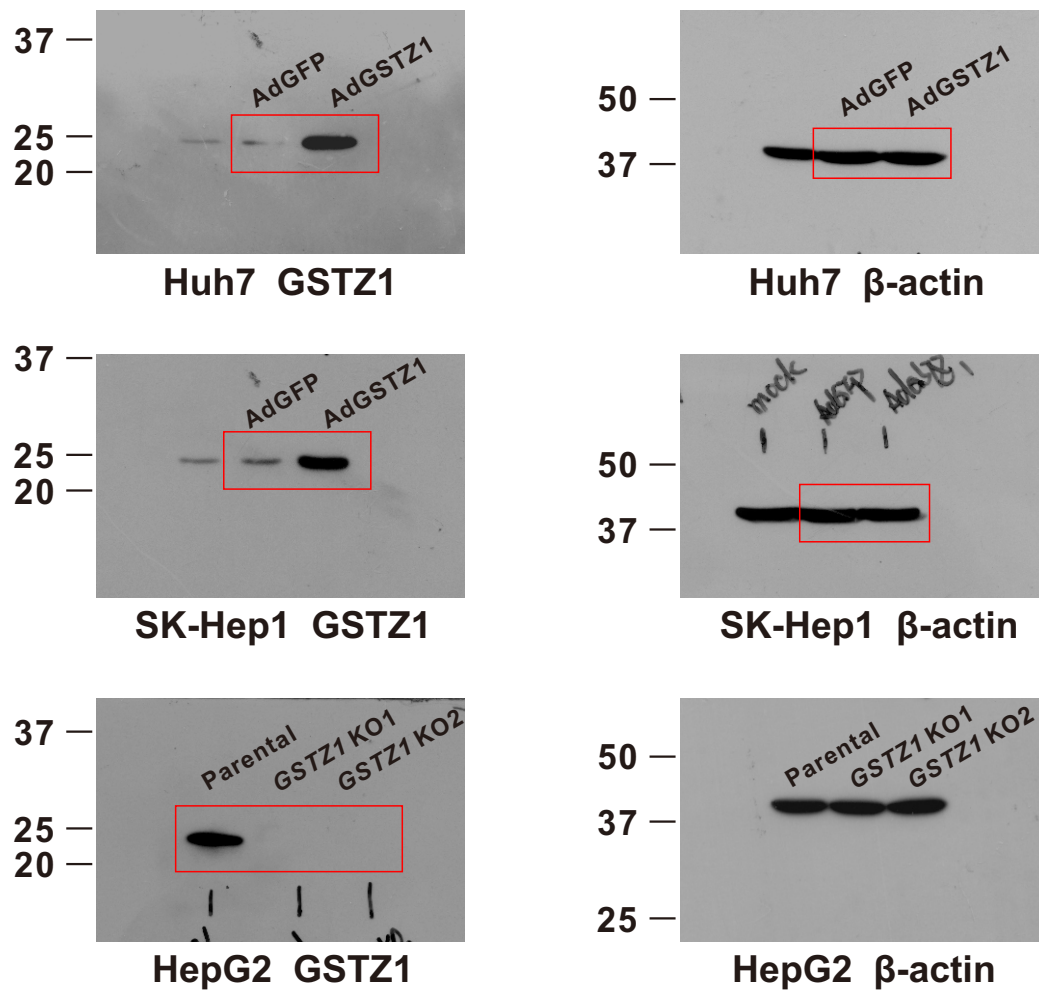

Supplement: Supplementary file 7 — Source Data for Figure 2 [file EMBJ-38-e101964-s006.zip › embj2019101964-sup-0006-SDataFig2/SourceDataGelsFig2.pdf]

**Fig.3C**

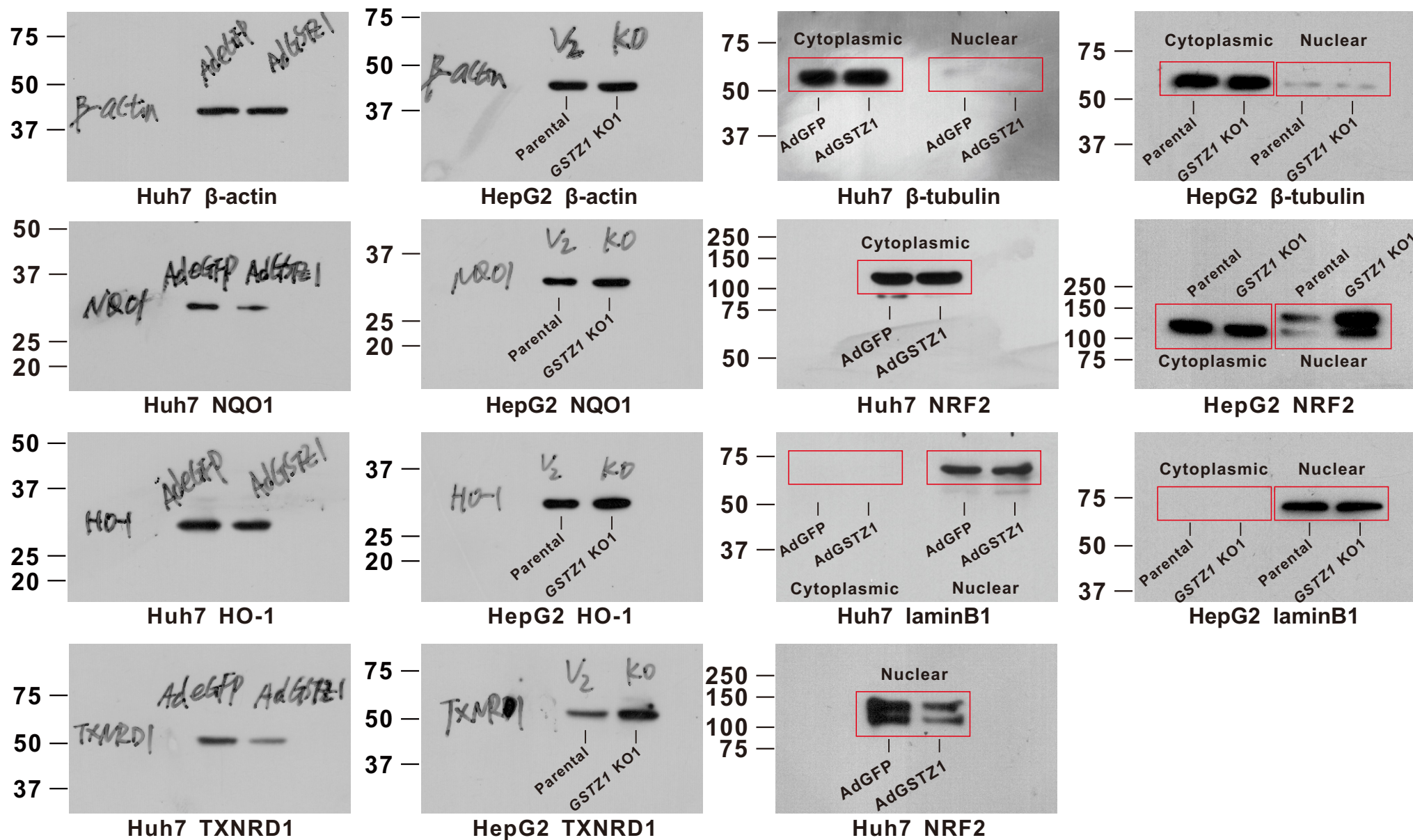

Fig.3F

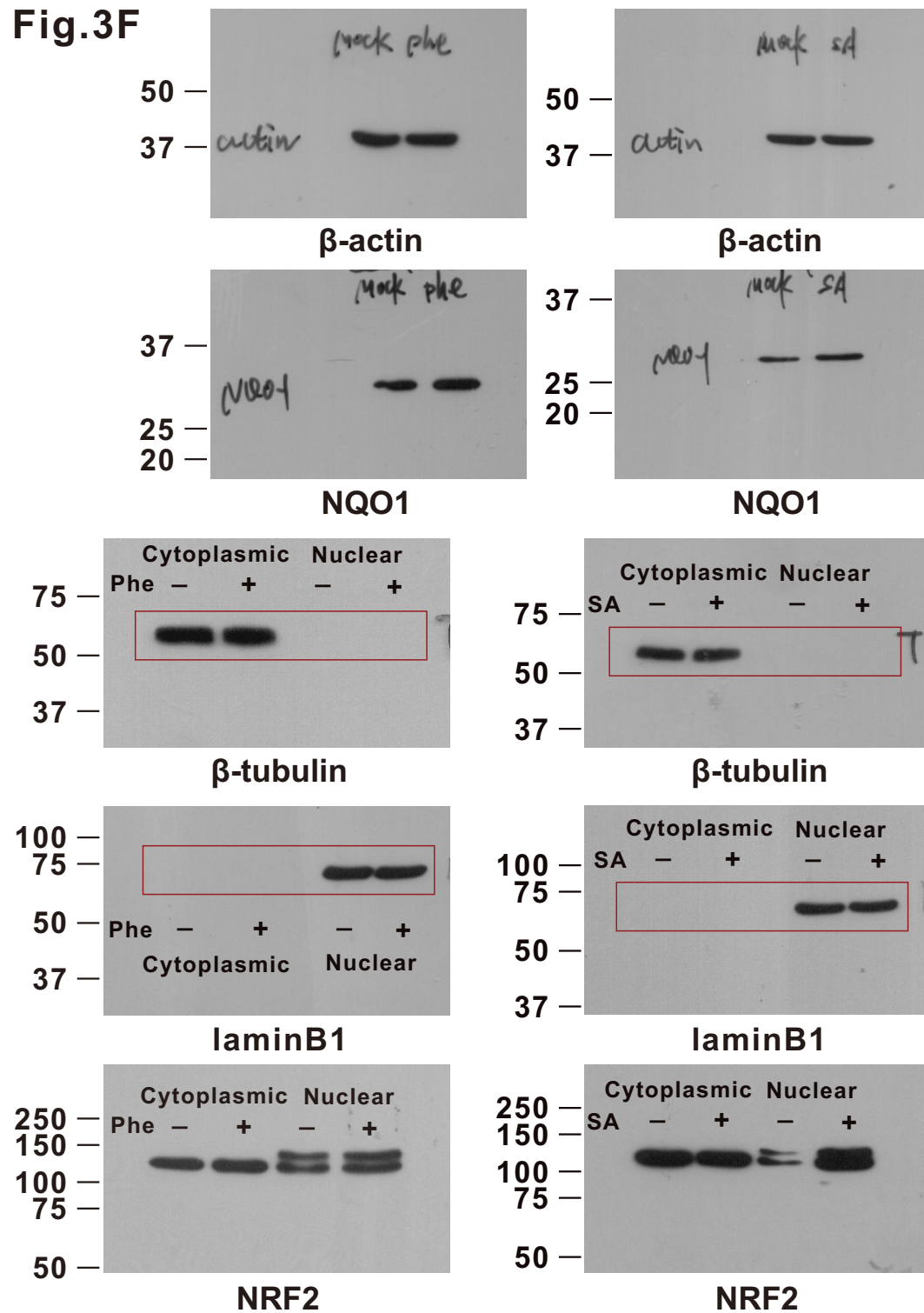

Fig.3H

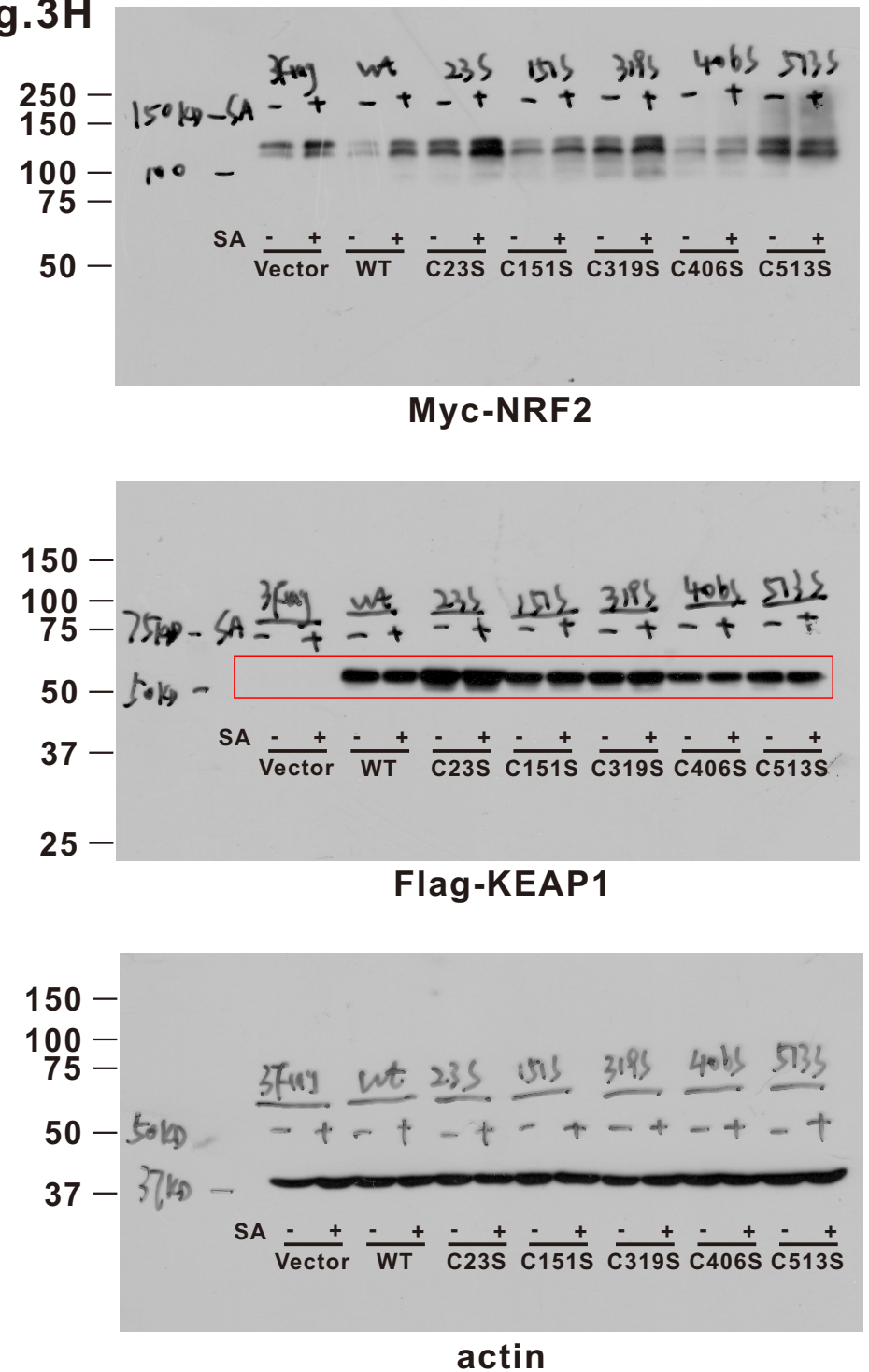

Supplement: Supplementary file 8 — Source Data for Figure 3 [file EMBJ-38-e101964-s007.zip › embj2019101964-sup-0007-SDataFig3/SourceDataGelsFig3.pdf]

**Fig. 4C**

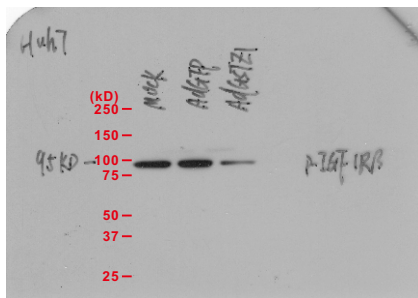

**Fig. 4C**

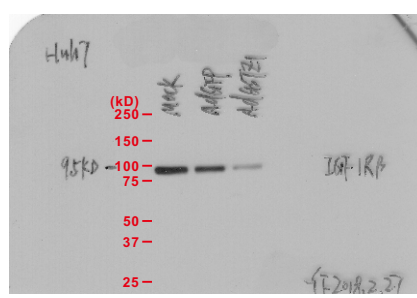

**Fig. 4C**

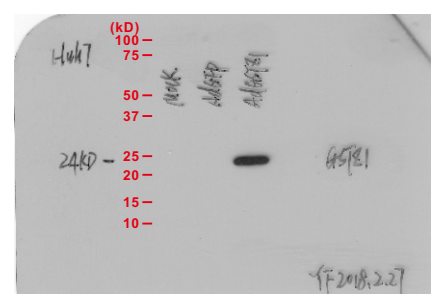

**Fig. 4C**

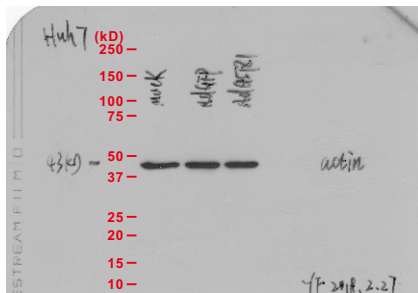

**Fig. 4C**

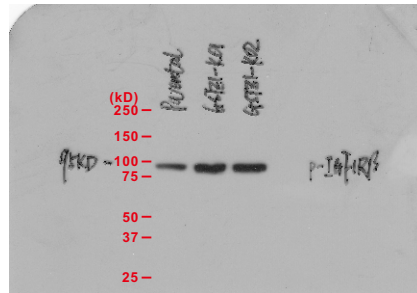

**Fig. 4C**

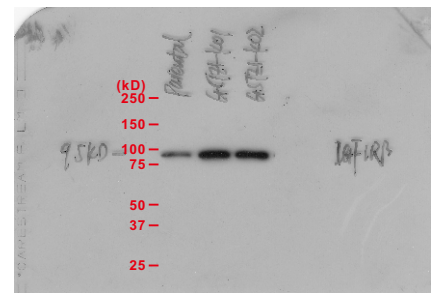

**Fig. 4C**

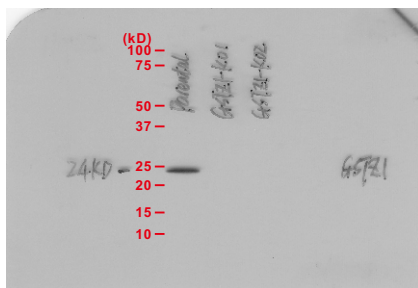

**Fig. 4C**

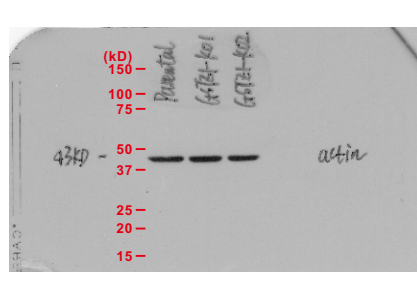

**Fig. 4E**

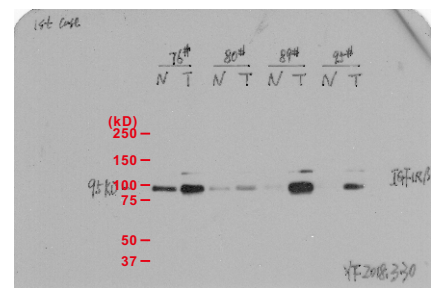

**Fig. 4E**

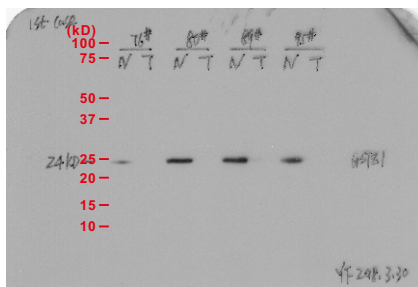

**Fig. 4E**

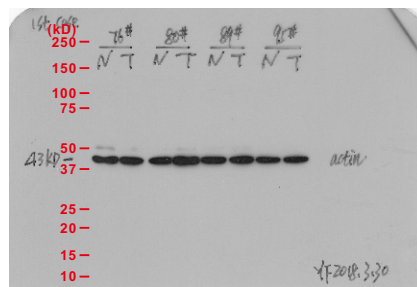

**Fig. 4E**

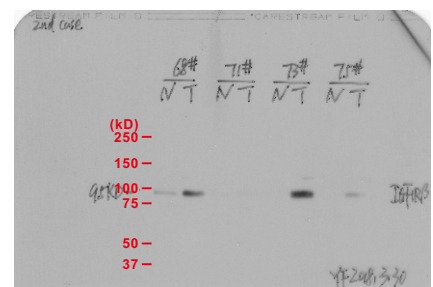

**Fig. 4E**

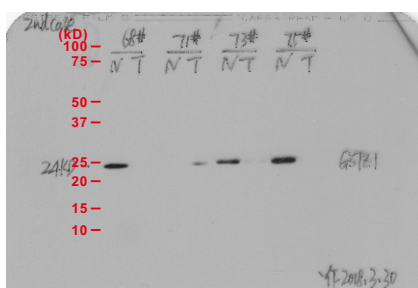

**Fig. 4E**

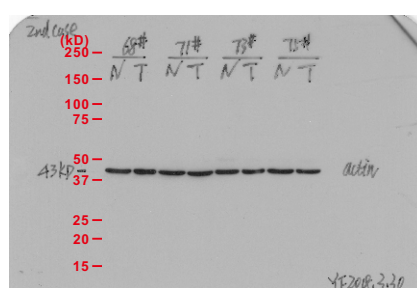

**Fig. 4E**

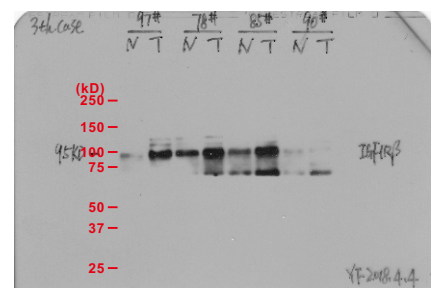

**Fig. 4E**

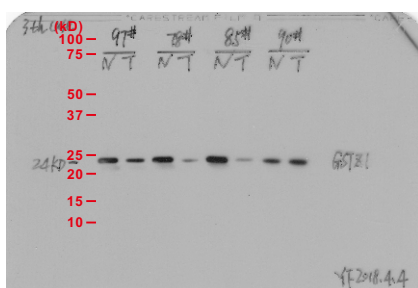

**Fig. 4E**

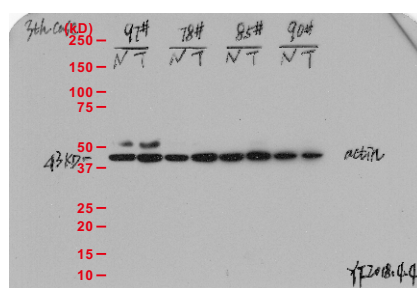

Supplement: Supplementary file 9 — Source Data for Figure 4 [file EMBJ-38-e101964-s008.zip › embj2019101964-sup-0008-SDataFig4/SourceDataGelsFig4.pdf]

Fig. 7D

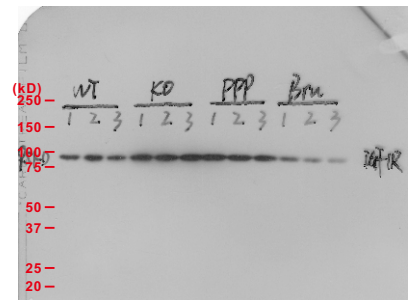

Fig. 7D

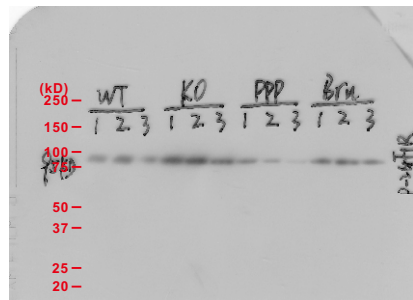

Fig. 7D

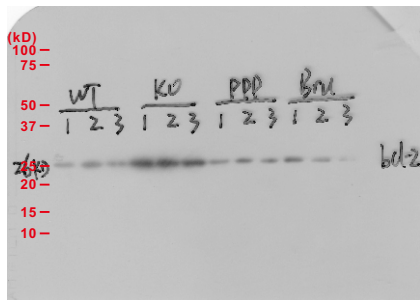

Fig. 7D

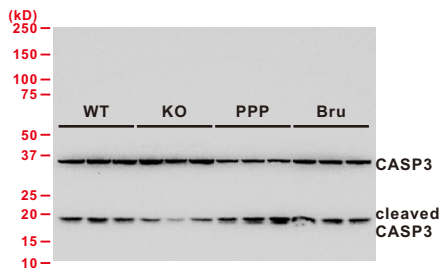

Fig. 7D

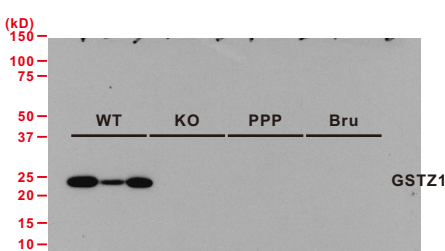

Fig. 7D

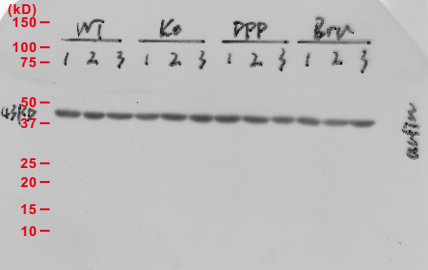

Supplement: Supplementary file 12 — Source Data for Figure 7 [file EMBJ-38-e101964-s011.zip › embj2019101964-sup-0011-SDataFig7/SourceDataGelsFig7.pdf]
