# Supplementary material for: GSTZ1‐1 Deficiency Activates NRF2/IGF1R Axis in HCC via Accumulation of Oncometabolite Succinylacetone
Source: EMBO J. 2019 Jun 28;38(15):e101964. doi: 10.15252/embj.2019101964 (PMC6669923; doi:10.15252/embj.2019101964)

Fig. 5F

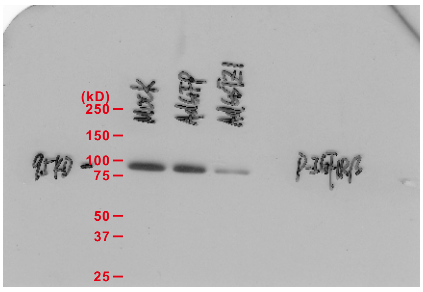

Fig. 5F

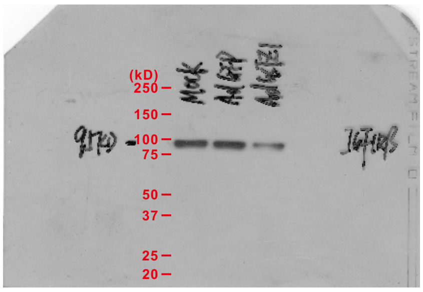

Fig. 5F

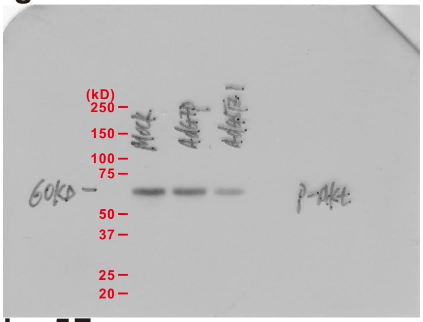

Fig. 5F

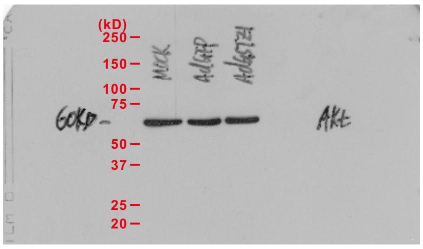

Fig. 5F

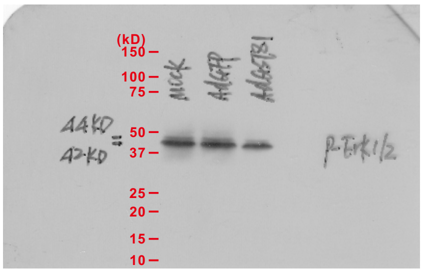

Fig. 5F

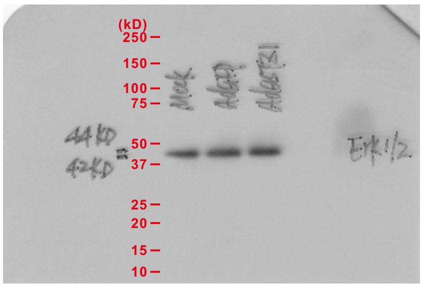

Fig. 5F

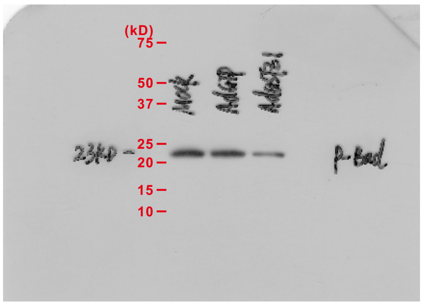

Fig. 5F

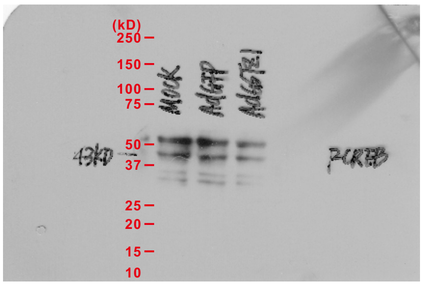

Fig. 5F

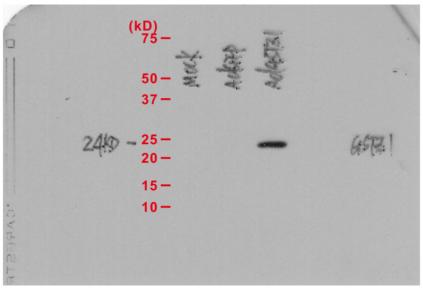

Fig. 5F

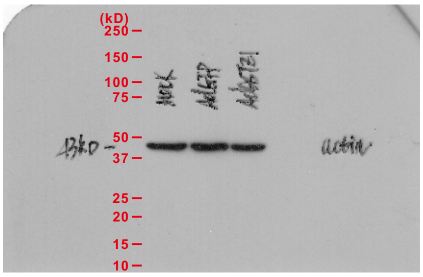

Fig. 5F

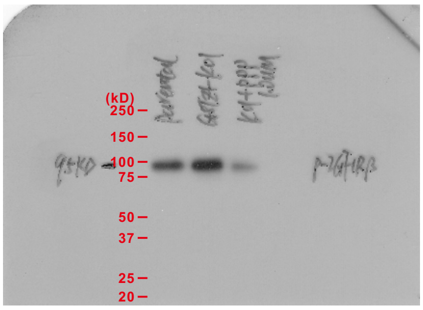

Fig. 5F

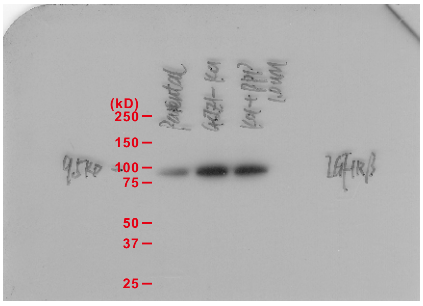

Fig. 5F

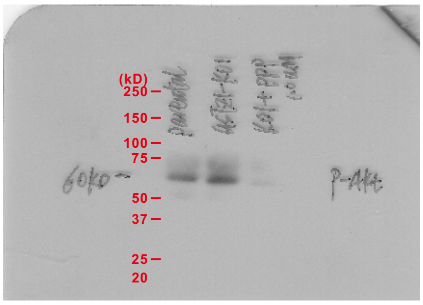

Fig. 5F

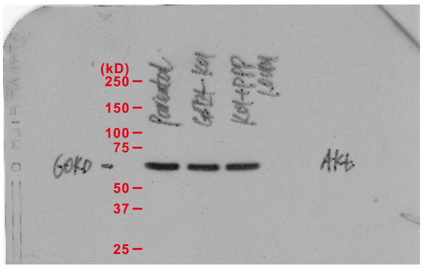

Fig. 5F

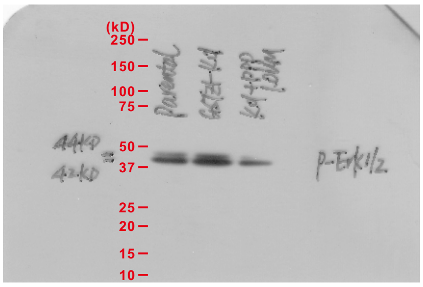

Fig. 5F

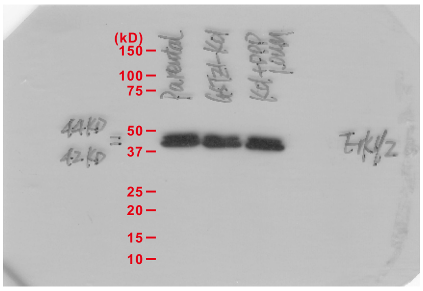

Fig. 5F

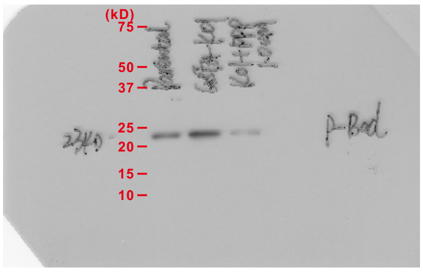

Fig. 5F

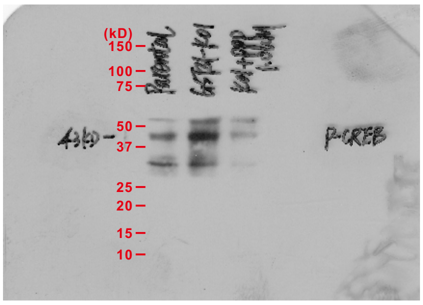

Fig. 5F

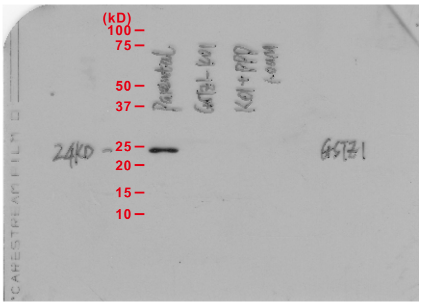

Fig. 5F

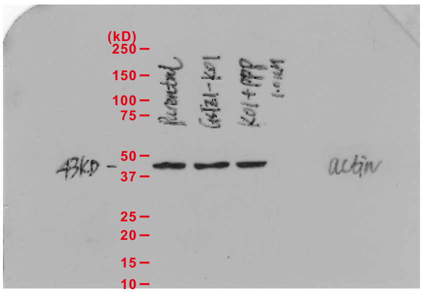

Fig. 5F

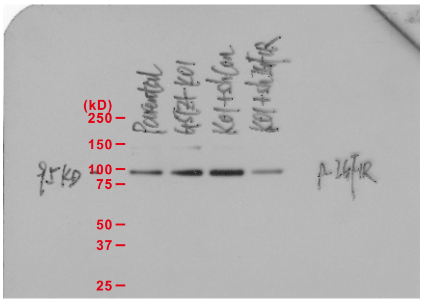

Fig. 5F

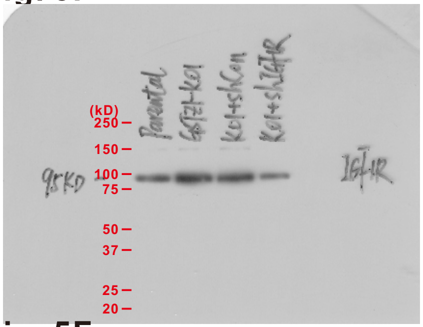

Fig. 5F

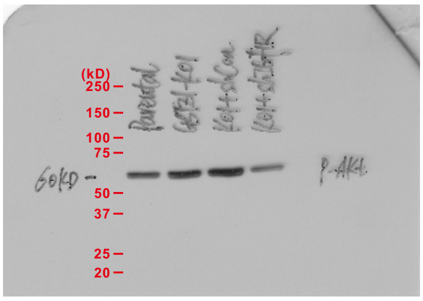

Fig. 5F

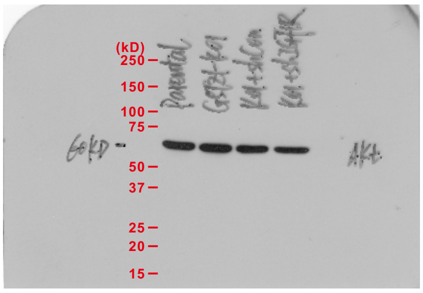

Fig. 5F

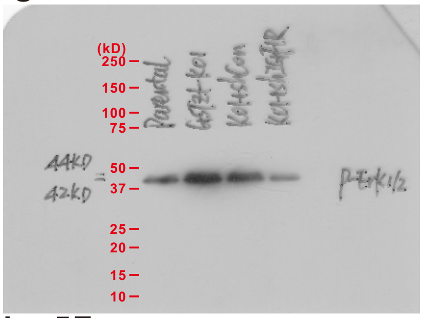

Fig. 5F

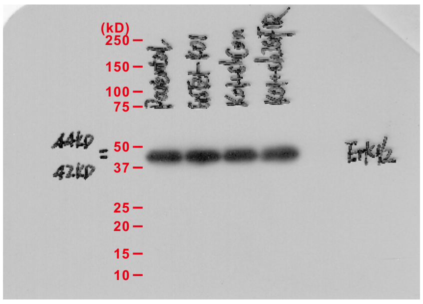

Fig. 5F

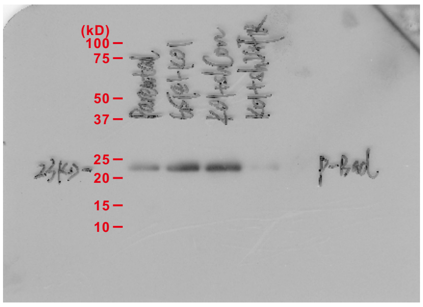

Fig. 5F

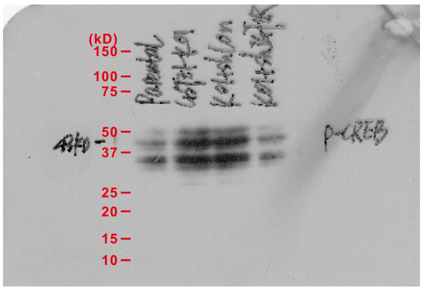

Fig. 5F

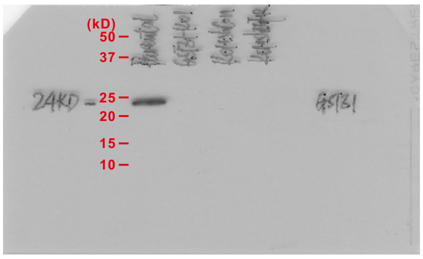

Fig. 5F

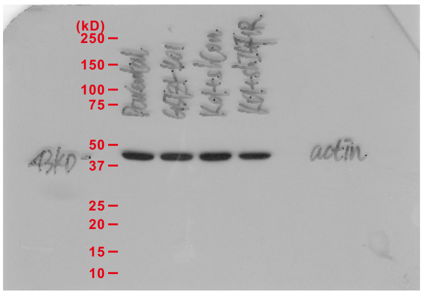

Fig. 5G

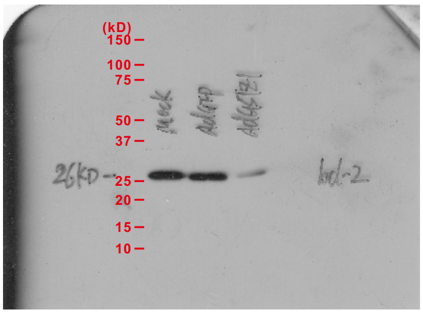

Fig. 5G

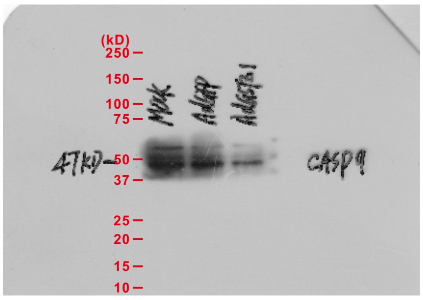

Fig. 5G

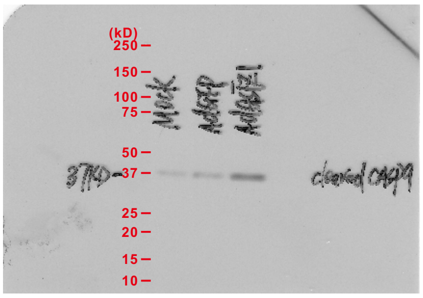

Fig. 5G

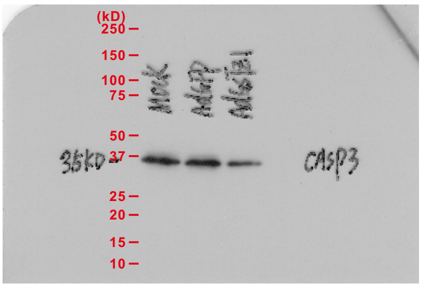

Fig. 5G

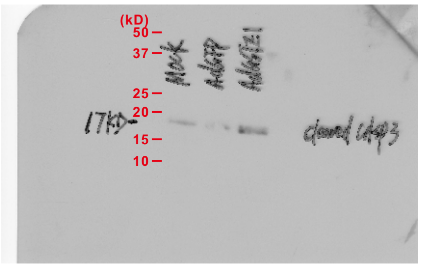

Fig. 5G

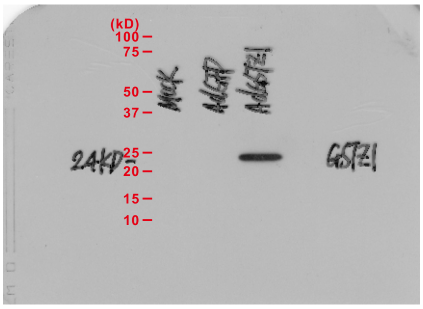

Fig. 5G

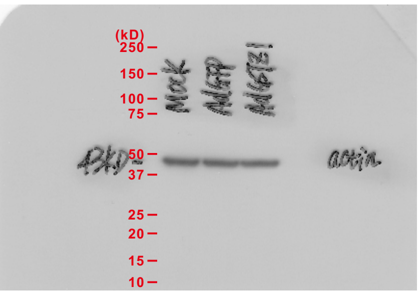

Fig. 5G

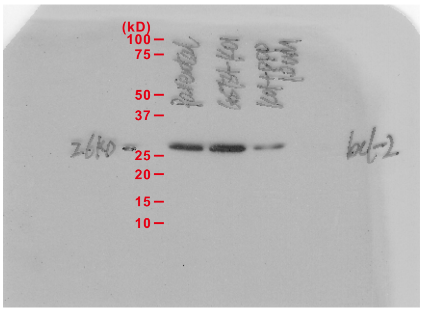

Fig. 5G

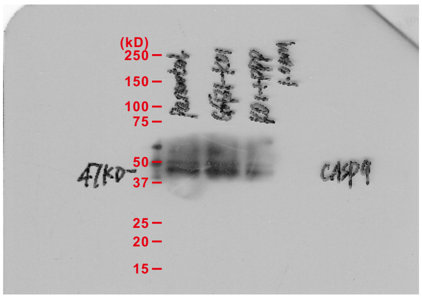

Fig. 5G

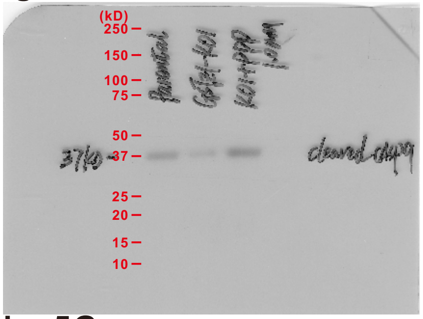

Fig. 5G

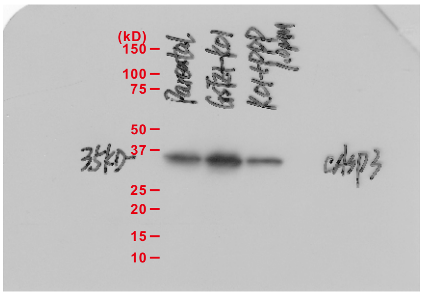

Fig. 5G

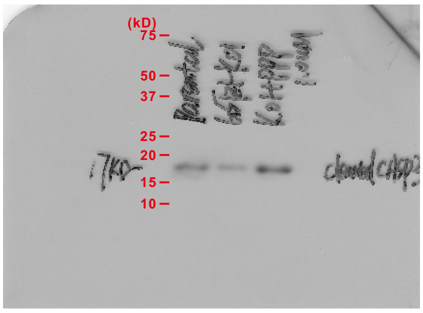

Fig. 5G

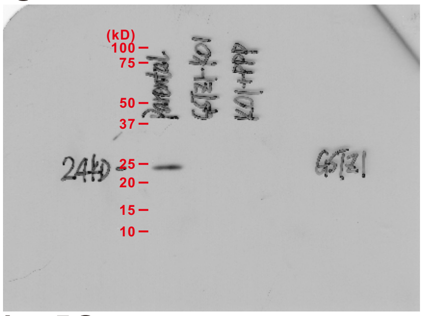

Fig. 5G

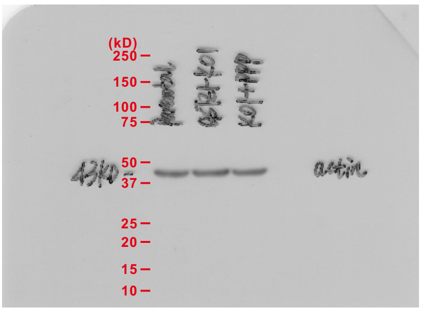

Fig. 5G

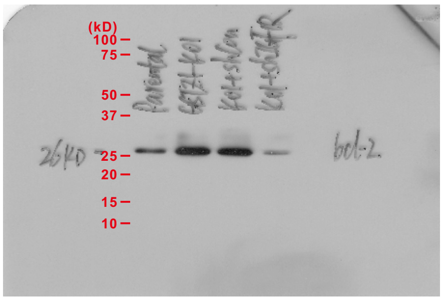

Fig. 5G

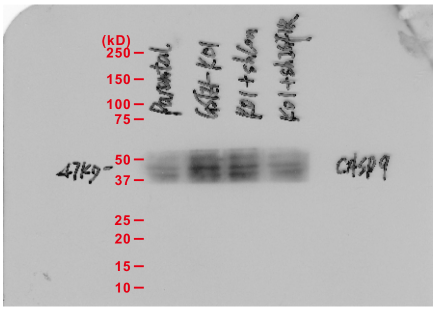

Fig. 5G

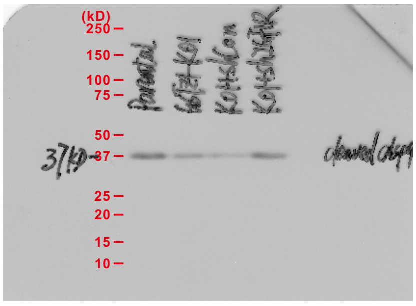

Fig. 5G

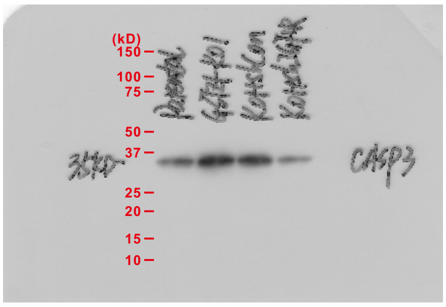

Fig. 5G

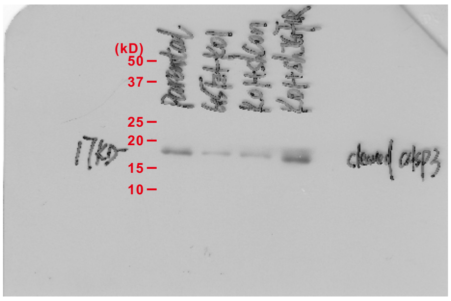

Fig. 5G

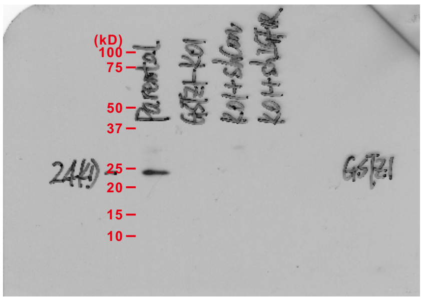

Fig. 5G

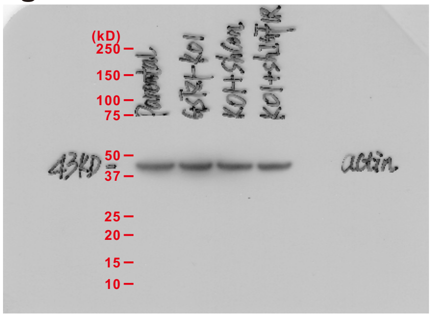

Supplement: Supplementary file 10 — Source Data for Figure 5 [file EMBJ-38-e101964-s009.zip › embj2019101964-sup-0009-SDataFig5/SourceDataGelsFig5.pdf]
