# Supplementary material for: GSTZ1‐1 Deficiency Activates NRF2/IGF1R Axis in HCC via Accumulation of Oncometabolite Succinylacetone
Source: EMBO J. 2019 Jun 28;38(15):e101964. doi: 10.15252/embj.2019101964 (PMC6669923; doi:10.15252/embj.2019101964)

Fig. 6A

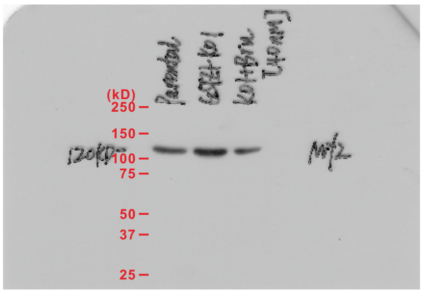


Fig. 6A

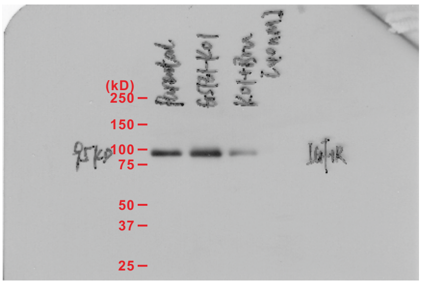


Fig. 6A

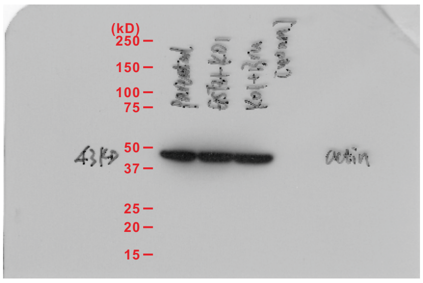


Fig. 6B

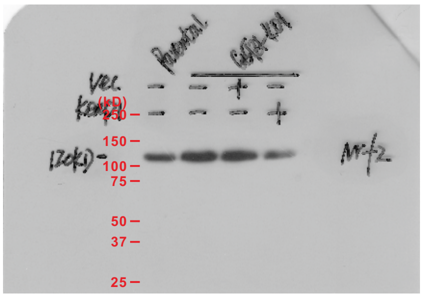


Fig. 6B

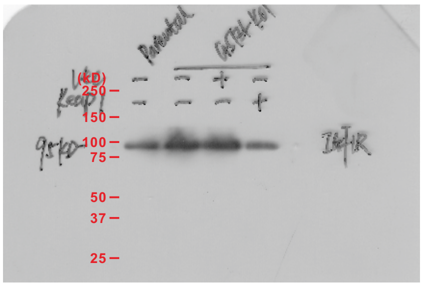


Fig. 6B

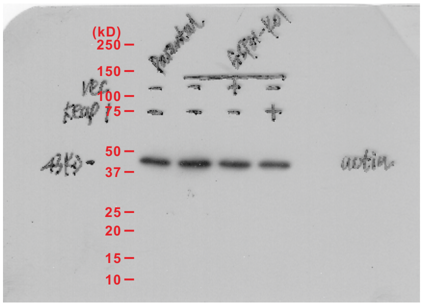


Fig. 6C

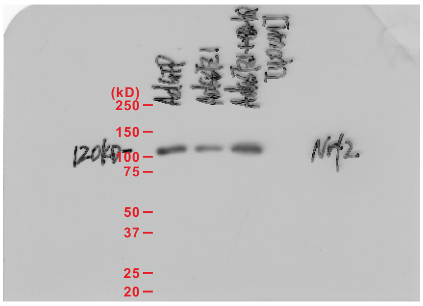


Fig. 6C

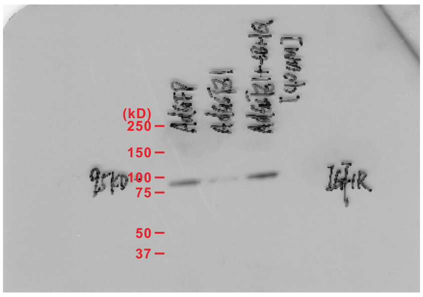


Fig. 6C

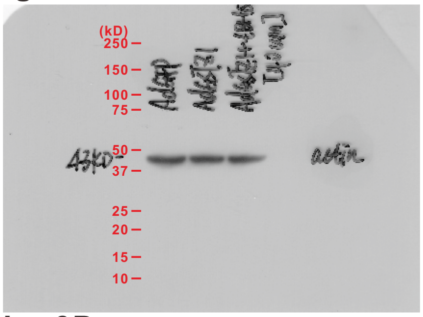


Fig. 6D

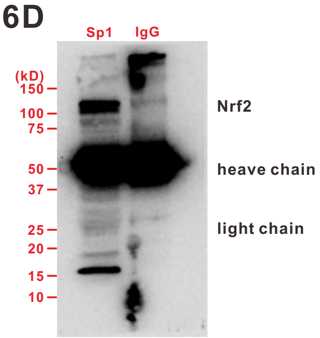


Fig. 6D

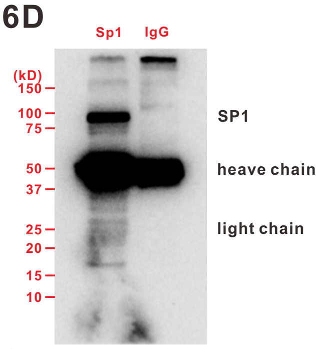


Fig. 6D

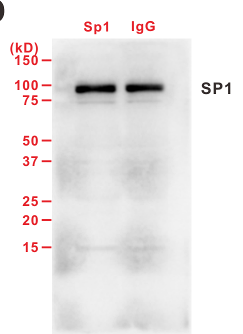


Fig. 6D

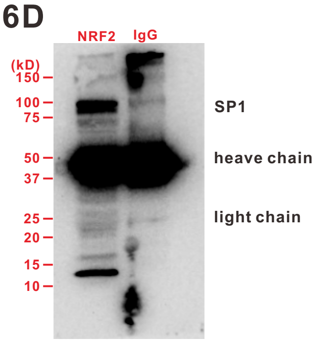


Fig. 6D

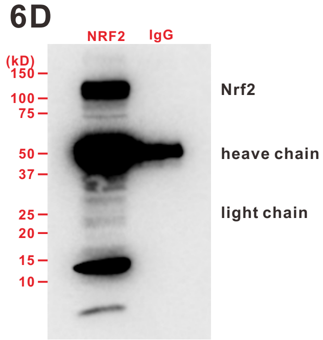


Fig. 6D

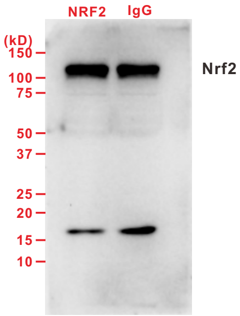


Fig. 6F

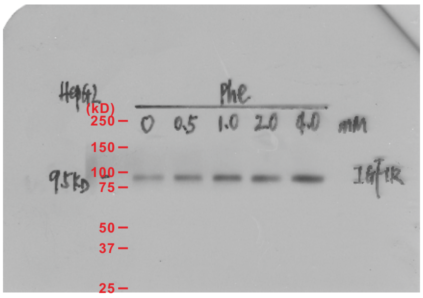


Fig. 6F

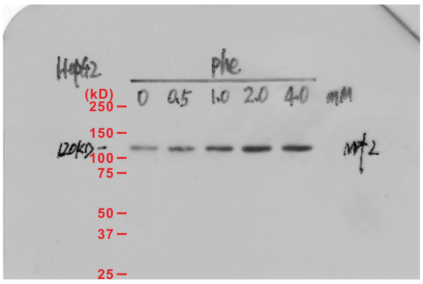


Fig. 6F

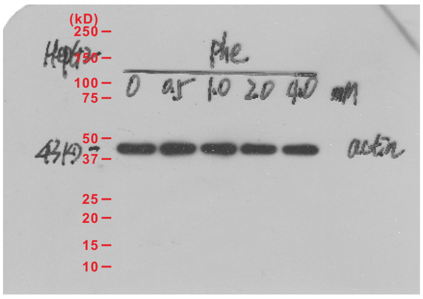

Fig. 6F

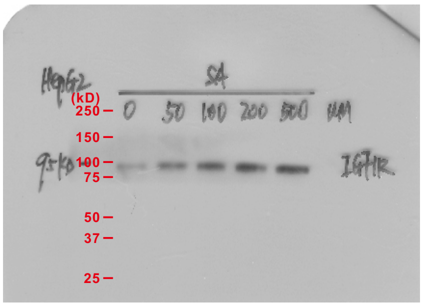


Fig. 6F

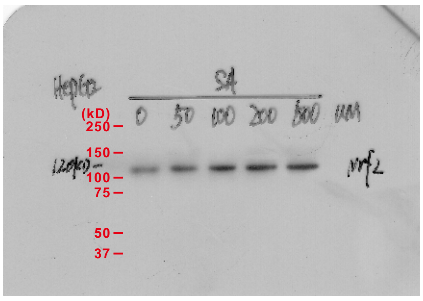


Fig. 6F

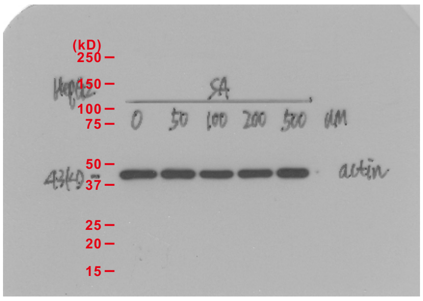


Fig. 6H

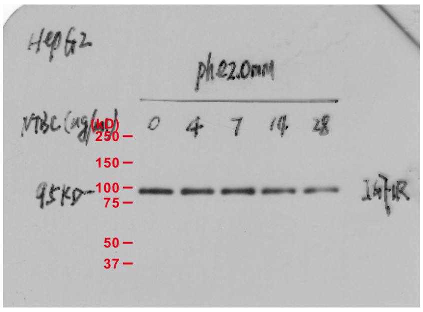


Fig. 6H

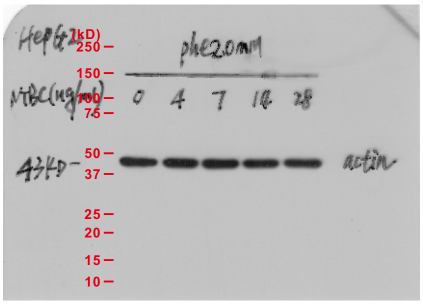


Fig. 6H

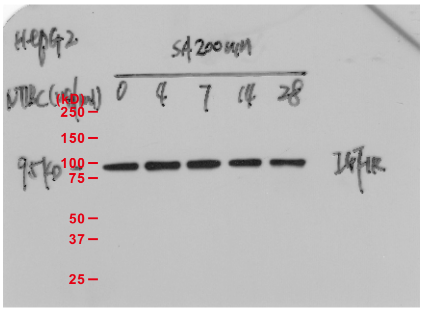


Fig. 6H

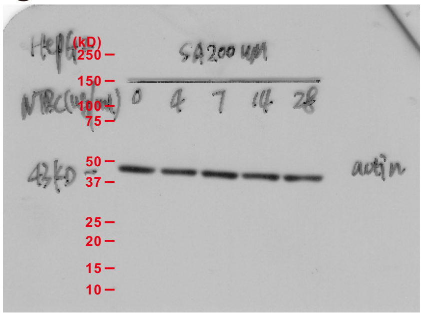

Supplement: Supplementary file 11 — Source Data for Figure 6 [file EMBJ-38-e101964-s010.zip › embj2019101964-sup-0010-SDataFig6/SourceDataGelsFig6.pdf]
